# Omega-6/Omega-3 Ratio as a Protective Factor in Lung Cancer: A Mendelian Randomization Study on Polyunsaturated Fatty Acids and Lung Adenocarcinoma Risk

**DOI:** 10.7150/jca.112416

**Published:** 2025-06-12

**Authors:** Cheng Wang, Ya Ding, Qikang Hu, Bin Wang, Shouzhi Xie, Zhi Yang, Zhe Zhang, Dexing Dai, An Xiong, Ruoman Sun, Yali Ling, Lei Qiu, Fenglei Yu, Zhongjian Xie, Muyun Peng

**Affiliations:** 1 The Second Xiangya Hospital of Central South University, Changsha, China.; 2 Hunan Key Laboratory of Early Diagnosis and Precise Treatment of Lung Cancer, Early-Stage Lung Cancer Center, Department of Thoracic Surgery, The Second Xiangya Hospital of Central South University, Changsha, China.; 3 Hunan Provincial Key Laboratory of Metabolic Bone Diseases, National Clinical Research Center for Metabolic Diseases, Department of Metabolism and Endocrinology, The Second Xiangya Hospital of Central South University, Changsha, China.

**Keywords:** polyunsaturated fatty acids, lung cancer, risk, mendelian randomization, causality

## Abstract

**Background:** While observational studies have reported conflicting associations between polyunsaturated fatty acids (PUFAs) and lung cancer risk, the causal role of specific PUFA subtypes remains unclear.

**Methods:** Leveraging genome-wide association data from the UK Biobank and International Lung Cancer Consortium, we employed univariable, multivariable, and bidirectional Mendelian randomization (MR) analyses to investigate the causal effects of seven PUFA traits (including omega-3, DHA, EPA, omega-6, LA, AA, and the omega-6/omega-3 ratio) on lung cancer and its subtypes.

**Results:** Our primary finding revealed a robust protective effect of a higher omega-6/omega-3 ratio against overall lung cancer (IVW: OR = 0.87; 95% CI: 0.78-0.96; *P_value* = 0.009) and lung adenocarcinoma (LUAD) (IVW: OR = 0.78; 95% CI: 0.67-0.89; *P_value* = 0.0005). Conversely, elevated omega-3 and DHA levels were associated with increased LUAD risk. These associations persisted after adjusting for BMI, smoking, and potential pleiotropy.

**Conclusion:** This study provides the first causal evidence that a higher omega-6/omega-3 ratio reduces lung cancer risk, particularly LUAD, through multivariable and bidirectional Mendelian randomization analyses that account for BMI, smoking, and genetic pleiotropy. These findings highlight the ratio's potential as a novel and modifiable dietary target for prevention, offering actionable insights beyond prior studies focused on individual PUFA subtypes.

## 1. Introduction

The impact of dietary fat on diseases has received increasing attention in recent decades. After digestion, fat is broken down into various fatty acids and glycerol. According to different structures, fatty acids are defined as saturated fatty acids and unsaturated fatty acids, of which unsaturated fatty acids are further divided into polyunsaturated fatty acids and monounsaturated fatty acids. Reasearchs have been obsearved that polyunsaturated fatty acids (PUFA) have vital roles in maintaining cellular function and homeostasis, including signal transduction, cell growth, differentiation and, maintaining cell viability 1. Due to the different positions of the first double bond at the methyl end of the carbon chain, we divide polyunsaturated fatty acids into two subtypes: omega-3 PUFA and omega-6 PUFA. Omega-3 PUFA includes docosahexaenoic acid (DHA) alpha-linolenic acid (ALA) and eicosapentaenoic acid (EPA), while the omega-6 PUFA includes arachidonic acid (AA) and linolenic acid (LA). Though the cytochrome P450 pathway (CYP450), the lipoxygenase pathway (LOX), and the cyclooxygenase pathway (COX) PUFA produces oxylipins, which are lipid derivates that mediate the action of PUFAs 2, 3. DHA and EPA are the precursors of anti-inflammatory oxidative lipids 4-6, whereas AA and LA are the precursors of pro-inflammatory oxidative lipids 7. Overall, polyunsaturated fatty acids play a crucial role in maintaining cellular homeostasis by mediating inflammatory responses 8, 9. Numerous studies have confirmed that disturbances in high intake or PUFA metabolism may lead to cancer risk and progression and contribute to cellular dysfunction 10-13.

Lung cancer ranks among the most frequently diagnosed cancers globally. According to the World Cancer Report 2020 (GLOBOCAN), there are approximately 2.2 million new lung cancer cases worldwide, representing about 11.4% of all cancer diagnoses, making it second only to breast cancer. Despite this, lung cancer remains the foremost cause of cancer-related mortality, with around 1.8 million deaths, constituting 18% of all cancer fatalities 14. Due to intra-tumor heterogeneity and resistance to anti-cancer drugs, the 5-year survival rate for lung cancer patients remains low at just 19% 15. Current treatments and therapies are insufficient to reduce mortality from this malignancy. Hence, prevention, especially a healthy diet and lifestyle, is crucial for lung cancer 16, 17. PUFA is an important nutrient associated with cancer, with potential anticancer effects that can be obtained from various food sources and incorporated into daily diet to maintain health 18. Numerous studies have explored the relationship between PUFAs and lung cancer risk, but the findings have been inconsistent. Certain studies suggest that PUFAs can inhibit lung cancer growth and metastasis by preventing the proliferation and migration of lung cancer cells 19-21. On the contrary, several other studies have shown that mice fed a diet rich in PUFAs increases lung cancer cell proliferation, angiogenesis, proinflammatory oxolipin, and cell invasion 22. In addition, clinical research data on the impact of PUFAs intake on lung cancer risk are inconsistent. Data from 10 cohorts by Yang et al. showed that high intake of PUFAs was associated with reduced risk of lung cancer 23. Data from the Shanghai Women's Health Study (SWHS) and Shanghai Men's Health Study (SMHS) indicated a negative correlation between the intake ratio of omega-6 to omega-3 PUFAs and lung cancer risk, while a positive correlation was observed with DHA intake 23. Conversely, a meta-analysis involving eight prospective cohort studies (including two from the U.S., two from Japan, and four from Europe) found no significant association between high PUFA intake and lung cancer risk 17. Therefore, the causal relationship between PUFAs and lung cancer remains ambiguous.

Mendelian randomization (MR) is a novel method that employs genetic variations, specifically single-nucleotide polymorphisms (SNPs), as instrumental variables to mimic the random assignment found in randomized controlled trials (RCTs). This approach provides evidence for hypothesized causal relationships between modifiable risk factors and diseases 24-26. Additionally, bidirectional MR effectively mitigates the issue of reverse causality that can bias traditional observational studies, as genotypes are established before disease onset and remain unaffected by disease progression 27, 28. To date, no MR studies have investigated the relationship between various polyunsaturated fatty acids and lung cancer or its subtypes. In this study, we opted to utilize univariable, multivariable, and bidirectional MR methods to uncover potential protective and risk factors for lung cancer.

## 2. Materials and Methods

In this research, adherence to the Strengthening the Reporting of Observational Studies in Epidemiology Using Mendelian Randomization (STROBE-MR) guidelines was maintained. Refer to Supplementary Materials 1 for the STROBE-MR Checklist Table 29.

### 2.1. Study design

**Figure 1:**
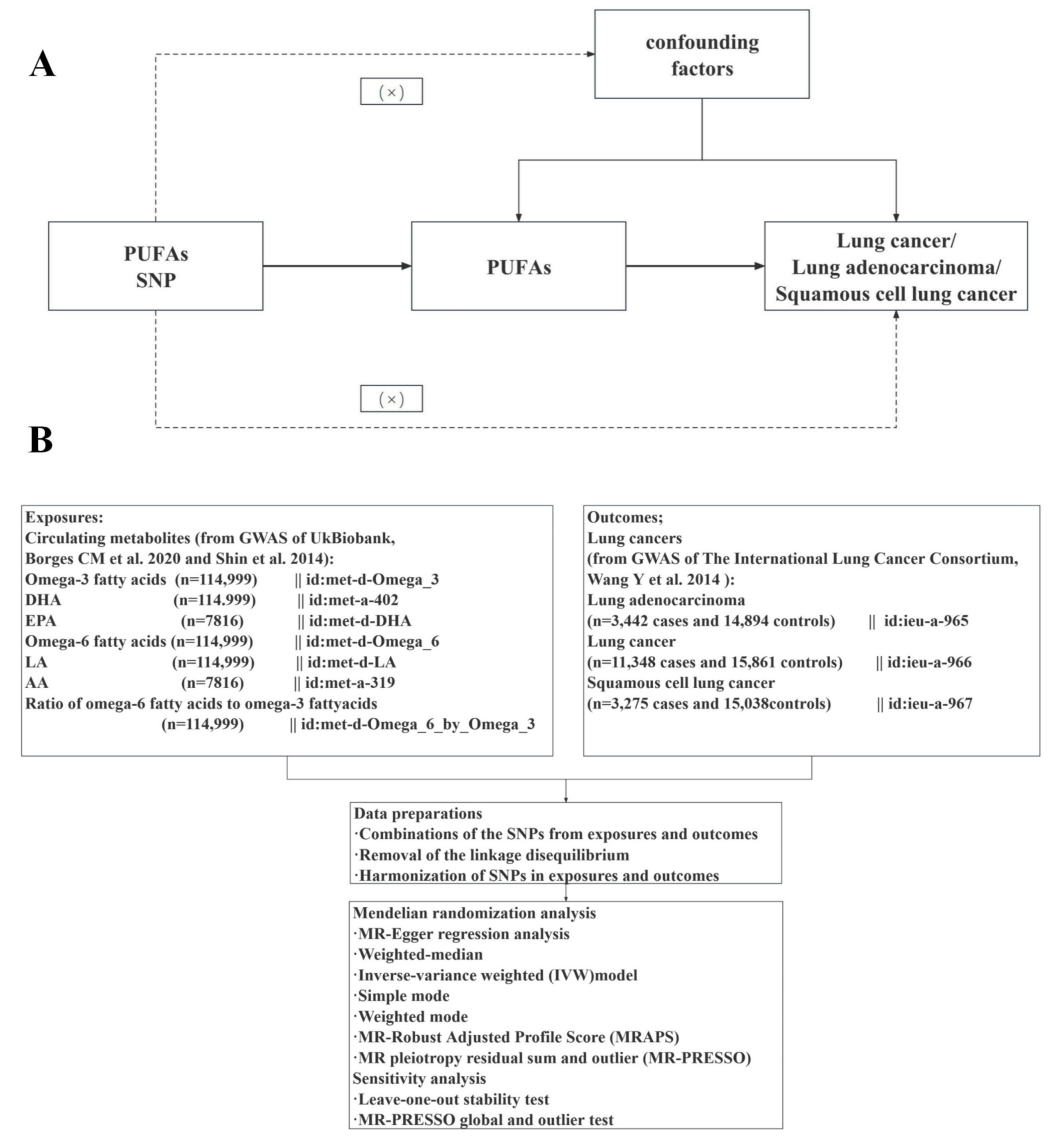
The overview flowchart of hypothesis and schematic design (A) Mendelian randomization key hypothesis Diagram. SNPs associated with PUFAs levels/ratios were used as the genetic instruments for investigating the causal effect of PUFA on lung cancer. The arrow lines indicate that the genetic instruments (SNPs) are associated with the exposure and can only affect the outcome via the exposure. The dashed lines indicate that the genetic instruments (SNPs) are independent of confounders between the results. (B) Schematic design for the mendelian randomization analysis.

Figure 1 illustrates the schematic overview of our hypothesis and design. Three core assumptions were established (Figure 1A) 30: (1) Genetic variations are closely associated with exposure, (2) Genetic variations are only related to the outcome through exposure, and (3) This relationship is not influenced by confounding factors. Publicly available data were employed, negating the need for additional informed consent or ethical approval. The genetic data were obtained from two extensive genome-wide association study (GWAS) datasets: UK Biobank and the International Lung Cancer Consortium (ILCCO). Following the exclusion of outliers and allele harmonization, five different methods were applied alongside sensitivity and heterogeneity analyses to investigate the causal links between PUFAs and lung cancer.

### 2.2. Data source and genetic instruments

Genetic instruments consist of one or more genetic variants, whose characteristics make them suitable as instrumental variables (IVs) in MR 31, 32. SNPs were identified from GWAS catalogs, IEU Open GWAS, and qualified datasets from NealELab, and used as IVs. To mitigate biases resulting from racial admixture, only GWAS conducted on individuals of European ancestry—defined as having at least 80% derived European ancestry—were included. IVs for MR analysis rely on meeting three essential assumptions. Genetic variants for MR analysis were selected based on the following criteria 27: (i) Single nucleotide polymorphisms (SNPs) had to be independent among instruments, assessed using the clumping algorithm with parameters set at r² = 0.001 and a 10,000 kb window; (ii) minor allele frequency (MAF) > 0.01; (iii) SNPs needed to have a P value below the genome-wide significance threshold in the respective data source; (iv) nonpalindromic SNPs (A/T or G/C polymorphisms with ambiguous allele frequencies) were excluded during the harmonization of exposure and outcome data. Furthermore, the average F-statistic of genetic instruments was estimated using the formula: *Fj = βj^2^/σj^2^* to evaluate instrument strength, where β and σ respectively represent the standard deviation of the estimated SNP exposure effect and its variation j. F-statistics were typically used to evaluate the risk of weak instrument bias, with a minimum value of 10 deemed sufficient for MR analysis 33, 34, thereby reducing the likelihood of weak instrument bias 30. Moreover, we used the following formula to estimate the proportion of explained variance in the association between genetic instruments and exposure variables: proportion of variance explained = *2β²ƒ(1-ƒ)*, where β represents the SNP exposure effect estimate and ƒ represents the MAF 35. Based on the merged exposure-outcome dataset, we conducted harmonization of effect alleles and subsequent analyses. Detailed information regarding IVs is provided in Supplementary Tables S1 - S7.

In this study, seven primary dietary PUFA indicators were examined, encompassing SNPs for levels of circulating omega-3, DHA, EPA, omega-6, LA, and AA, along with the omega-6 to omega-3 fatty acid ratio. Genetic variants related to exposure were sourced from metabolic biomarkers in the UK Biobank (Nightingale Health, 2020), specifically for omega-3, omega-6, DHA, LA, and the omega-6/omega-3 ratio. Omega-3 and omega-6 fatty acids, as well as DHA and LA concentrations, were assessed in randomly selected EDTA plasma samples using a specialized high-throughput nuclear magnetic resonance metabolomics platform (Nightingale Health Ltd; BioMarker Qualification Version 2020) 36. After removing duplicate entries and those that failed quality control from non-fasting plasma samples collected at baseline, 121,577 samples were initially kept for analysis, which resulted in 114,999 usable samples. The specifics of the measurement technology used in this platform and its applications in epidemiology have been documented in earlier studies 37, 38. SNPs linked to omega-3, omega-6, DHA, LA, and the omega-6/omega-3 ratio (n=114,999) were identified as genetic instruments for each phenotype using conventional GWAS significance thresholds (P < 5 ×10^-8^). The serum samples in this study were sourced from the Twins United Kingdom cohort 39, a registry of adult twins in the UK, predominantly women, recruited from the general population through national media. The serum levels of EPA and AA were measured using the Metabolon platform, with specific details thoroughly described in previous studies 33, 40, 41. Additionally, summary statistics of SNPs associated with EPA and AA (n=7,816) with genome-wide significance (P < 5 × 10^-6^) were designated as alternate IVs. Genetic data concerning lung cancer and its pathological subtypes were sourced from the ILCCO 42, 43. The ILCCO consortium provided its aggregated data on the MR-Base platform, encompassing 27,209 individuals of European ancestry (11,348 with lung cancer and 15,861 controls). Among the lung cancer cases, 3,275 were classified as squamous cell lung cancer (SQLC), and 3,442 were classified as lung adenocarcinoma (LUAD).

### 2.3. Mendelian randomization

Separate two-sample univariate MR analyses were conducted to assess the causal effects of each PUFA (omega-3, DHA, EPA, omega-6, LA, AA, and omega-6/omega-3) on overall lung cancer and its two main pathological types (adenocarcinoma and squamous cell carcinoma). The primary analysis used the inverse variance weighted (IVW) method 44, which combined the Wald ratio estimates of the causal effects from various variants under the assumption that all IVs were valid. To address pleiotropy, we also applied four additional MR methods that relax the IV assumptions: MR-Egger regression 45, weighted median 46, simple mode and weighted mode 47 methods. Reciprocal MR analyses were performed to estimate the causal effects of overall lung cancer and its two main pathological types (adenocarcinoma and squamous cell carcinoma) on the PUFAs separately. This approach also helps determine the correct direction of causality. Cochran's Q test 48 and MR-Egger intercept test 49 were utilized to detect heterogeneity and horizontal pleiotropy, respectively. To address potential violations of MR assumptions due to directional pleiotropy, MR-Egger regression analysis and weighted-median estimates were applied 45, 46.

Additionally, MR-PRESSO was employed for identifying and addressing horizontal pleiotropy by excluding outliers with a P-value < 0.05, thereby obtaining a refined estimate of the causal effect 50. Heterogeneity was assessed using Cochran's Q-statistic, and subsequent analyses were conducted using a random-effects model with a significance level of P-value < 0.05 for substantial heterogeneity detection 51. During MR-PRESSO analysis, heterogeneity and pleiotropy in causal effect estimates were mitigated by eliminating outliers and reassessing the causal estimates. If significant heterogeneity persisted after outlier removal, all SNPs with a P-value < 1 in the MR-PRESSO outlier test were excluded. The MR analysis was conducted, adopting results from the random-effect IVW model. The number of distributions in the MR-PRESSO analysis was set to 10,000. Additional sensitivity analyses involved the exclusion of IVs one at a time 52. Other statistical tools were utilized to complement IVW, resulting in broader confidence intervals (CIs) 53. Consequently, IVW results were prioritized, with MR-Egger employed for significant pleiotropy and MR-PRESSO used for final outlier detection. The flowchart detailing the analytical methods used in this MR analysis is depicted in Supplementary Figure 1.

In further analyses to explore the direct effects of PUFAs on lung cancers, a multivariable MR (MVMR) analysis was conducted, extending the univariable MR to jointly detect causal effects of multiple risk factors 54, 55. GWAS summary data for body mass index (BMI) 56 and smoking-related traits were gathered from large-scale GWAS or relevant meta-analyses. The BMI data were sourced from the meta-analysis of UK Biobank and Genetic Investigation of Anthropometric Traits (GIANT) consortium (https://portals.broadinstitute.org/collaboration/giant/index.php/GIANT_consortium_data_files), encompassing 681,275 European individuals. Smoking-related SNP effect sizes were derived from the GWAS pipeline using Phesant variables from UK Biobank, which included current tobacco smoking (n = 462,434), smoking/smokers in the household (n = 425,516), and past tobacco smoking (n = 424,960). These datasets (Supplementary Table S9) are available for downloaded at the IEU Open GWAS database (https://gwas.mrcieu.ac.uk/).

MVMR accounts for the relationships among PUFAs, BMI, and smoking, recognizing that SNPs used in MR analyses often associate with multiple phenotypes. Since BMI and smoking are related to lung cancer development 57, 58, MVMR was utilized to assess the direct effects of PUFAs, independent of BMI and smoking influences. To reduce the impact of strong LD in all mediators, a clumping window of r2 = 0.001 and kb = 10,000 was applied. All GWAS-significant SNPs with a P-value less than 5 × 10^-8^ from each exposure were extracted and clumped to avoid LD within a window of r2 = 0.001 and kb = 10,000. The selected IVs were then analyzed using the multivariable IVW model, with a P-value < 0.05 deemed significant in the MVMR analysis.

### 2.4. Statistical analysis

The odds ratios (ORs) and corresponding 95% CIs were employed to evaluate the strength of causal associations. All *P*_values were two-sided, with a conventional significance threshold set at *P*_value <0.05. MR analyses and clumping were conducted using the“TwoSampleMR” 59 package (version 0.5.6) and “Mendelian Randomization” 60 packages (version 0.5.0) in R software (version 4.3.2), R Foundation for Statistical Computing, Vienna, Austria).

## 3. Results

Data on SNPs related to PUFA exposures (including omega-3, DHA, EPA, omega-6, LA, AA, and the omega-6/omega-3 ratio) are detailed in Supplementary Tables S1-7. All selected instrumental variables (IVs) had F-statistics greater than 10, indicating that there were no weak genetic instruments. Sensitivity analysis results and identified outliers are summarized in Table 2.

### 3.1. Causal effects of Omega-3, DHA and EPA on lung cancers

Univariate MR analyses demonstrated that higher genetically predicted levels of omega-3 (IVW: OR :1.24; 95% CI: 1.08 to 1.42; *P*_value = 0.021) and DHA (IVW: OR: 1.34; 95% CI: 1.13 to 1.59; *P*_value = 0.0027) were associated with an increased risk of LUAD. Additionally, DHA (IVW_adjusted: OR: 1.15; 95% CI: 1.05 to 1.27; *P*_value = 0.0027) was linked to an elevated risk of overall lung cancer. However, no associations were found between omega-3, DHA, or EPA and SQLC, nor between EPA and any lung cancer types.

#### 3.1.1. Causal effects of Omega-3 on lung cancers

Regarding the association of omega-3 with overall lung cancer and SQLC risk, there was no evidence to suggest directional pleiotropy but significant heterogeneity was oberved, as demonstrated by Cochran's Q test (lung cancer: Q = 82.5417, *P*_value = 0.0002; SQLC: Q = 77.4377, *P*_value=0.0007) (Table 2). Removal of three outliers (rs139974673, rs174564, rs2394976) eliminated heterogeneity in the analysis of omega-3 and SQLC, but no relationship with SQLC was found (Table 1). After removal of two outliers (rs174564, rs2394976), heterogeneity persisted in the analysis of omega-3 and overall lung cancer (Q_adjusted = 56.1368, *P*_value_adjusted= 0.0466) (Table 2). This heterogeneity may be attributed to data originating from different analysis platforms. To mitigate its impact, MR analysis was conducted using results of a random effect IVW model, and there was still no significant association between omega-3 and overall lung cancer (Table 1). No directional pleiotropy or heterogeneity was found between omega-3 in the circulation and LUAD, and the genetically determined omega-3 was associated with increased risk of LUAD (IVW: OR :1.24; 95% CI: 1.08 to 1.42; *P*_value = 0.021) (Table 2).

#### 3.1.2. Causal effects of DHA on lung cancers

The association of DHA on overall lung cancer, LUAD and SQLC showed heterogeneity respectively, detected by Cochran's Q test (lung cancer: Q = 56.9144, *P*_value = 0.0192; LUAD: Q = 53.9483, *P*_value=0.0355; SQLC: Q = 56.4246, *P*_value=0.0213), but no directional pleiotropy (Table 2). Removal of one outlier (rs2394976) in the analysis of DHA and overall lung cancer eliminated heterogeneity, and a significant association emerged (IVW_adjusted: OR: 1.15; 95% CI: 1.05 to 1.27; *P*_value = 0.0027), but after removal of three outliers (rs139974673, rs174564, rs2394976) eliminated heterogeneity, there was still no significant association of DHA and SQLC (Table 1). However, there was no outliers in the analyses of the association between DHA and LUAD. Despite heterogeneity, considering that our MR analysis used an IVW random effects model, the findings regarding the association between DHA and an increased risk of LUAD (IVW-random: OR: 1.34; 95% CI: 1.13 to 1.59; *P*_value = 0.0027) are still valid, (Table 1).

#### 3.1.3. Causal effects of EPA on lung cancers

No significant heterogeneity was observed in the analysis of EPA levels and lung cancer, including its pathological subtypes. Assessment of pleiotropy through MR-Egger regression revealed intercept = -0.0646 (*P*_value = 0.0495) for lung cancer and intercept = -0.1011 (*P*_value = 0.0499) for SQLC (Table 2). Despite the removal of two outliers (rs174556, rs179976) and one outlier (rs174556) respectively, no significant association was found in both analyses (Table 1). Furthermore, no evidence of directional pleiotropy or association was found in the analysis of EPA levels and LUAD (Table 1).

### 3.2. Causal effects of Omega-6, LA, AA and the ratio of omega-6 to omega-3 on lung cancers

No significant correlation was found for the analyses of LA and AA on lung cancer and its pathological subtypes except for omega-6 and omega-6/omega-3. Univariate MR analysis revealed that higher genetically predicted level of omega-6 (IVW_adjusted: OR :1.26; 95% CI: 1.05 to 1.51; *P*_value = 0.0126) was associated with an increased risk of LUAD. In addition, protective associations were observed in the analyses of omega-6/omega-3 on overall lung cancer (IVW_adjusted: OR: 0.87; 95% CI: 0.78 to 0.96; *P*_value = 0.0090) and LUAD (IVW: OR: 0.78; 95% CI: 0.67 to 0.89; *P*_value = 0.0005). However, there was no evidence of an association between omega-6 or omega-6/omega-3 and SQLC, while omega-6 showing no correlation with overall lung cancer in our study.

#### 3.2.1. Causal effects of Omega-6 on lung cancers

The associations between omega-6 and overall lung cancer and its pathological subtypes all showed heterogeneity, detected by Cochran's Q test (lung cancer: Q = 91.9865, *P*_value = 9.6233e-05; LUAD: Q = 76.5984, *P*_value = 0.0041; SQLC: Q = 73.6822, *P*_value = 0.0077), but no directional pleiotropy (Table 2). In the analyses of omega-6 on overall lung cance and SQLC, after removal of two outliers (rs28383314, rs79429216) and one outlier (rs79429216), we found that heterogeneity was eliminated in both analyses, but no significant associations were found either. However, after removal of four outliers (rs1002687, rs5754102, rs28383314, rs79429216), a significant association was only revealed between Omega-6 and increased risk of LUAD (Table 1).

#### 3.2.2. Causal effects of LA on lung cancers

The association of LA with overall lung cancer and its pathological subtypes all showed no evidence of directional pleiotropy but significant heterogeneity, according to Cochran's Q test (lung cancer: Q = 77.9955, *P*_value = 0.0003; LUAD: Q = 62.6224, *P*_value = 0.0126; SQLC: Q = 63.9807, *P*_value = 0.0094) (Table 2). Removing outliers eliminated heterogeneity, but there was still no significant association of LA with overall lung cancer and its pathological subtypes (Table 1).

#### 3.2.3. Causal effects of AA on lung cancers

No heterogeneity or directional pleiotropy was found for the analyses of AA on overall lung cancer and LUAD (Table 2), and no association was found between AA and overall lung cancer and LUAD (Table 1). The association of AA with SQLC risk showed no evidence of directional pleiotropy but significant heterogeneity, according to Cochran's Q test (Q = 20.3167, *P*_value = 0.0161) (Table 2), but no outlier was found after MR-PRESSO detected. Using the random-effect IVW model, circulating AA was not found to be significantly associated with SQLC (Table 1).

#### 3.2.4. Causal effects of the ratio of omega-6 to omega-3 on lung cancers

The association of omega-6/omega-3 with overall lung cancer and its pathological subtypes risk showed no evidence of directional pleiotropy, but significant heterogeneity was found for the analyses of omega-6/omega-3 on overall lung cancer and SQLC except for LUAD, according to Cochran's Q test (lung cancer: Q = 72.4508, *P*_value = 4.9808e-06; SQLC: Q = 63.9807, *P*_value = 0.0003) (Table 2). After removal of three outliers (rs139974673, rs174564, rs2394976), we found that heterogeneity was eliminated in the analysis of omega-6/omega-3 on SQLC, but no significant associations were found either. While removal of one outlier (rs2394976), heterogeneity (lung cancer: Q-adjusted = 72.4508, *P*_value_-adjusted_ = 4.9808e-06) (Table 2) was still found in the analysis of omega-6/omega-3 on overall lung cancer. Using the random-effect IVW model, circulating omega-6/omega-3 was found to be significantly associated with a decreased risk of overall lung cancer (IVW-random OR: 0.87; 95% CI: 0.78 to 0.96; *P*_value = 0.0090). And a suggestively significant association between omega-6/omega-3 and lower risk of LUAD (IVW: OR:0.78; 95% CI: 0.67 to 0.89; *P*_value = 0.0005) was also found.

### 3.3. Results of multivariable MR and bidirectional MR analyses

**Figure 2:**
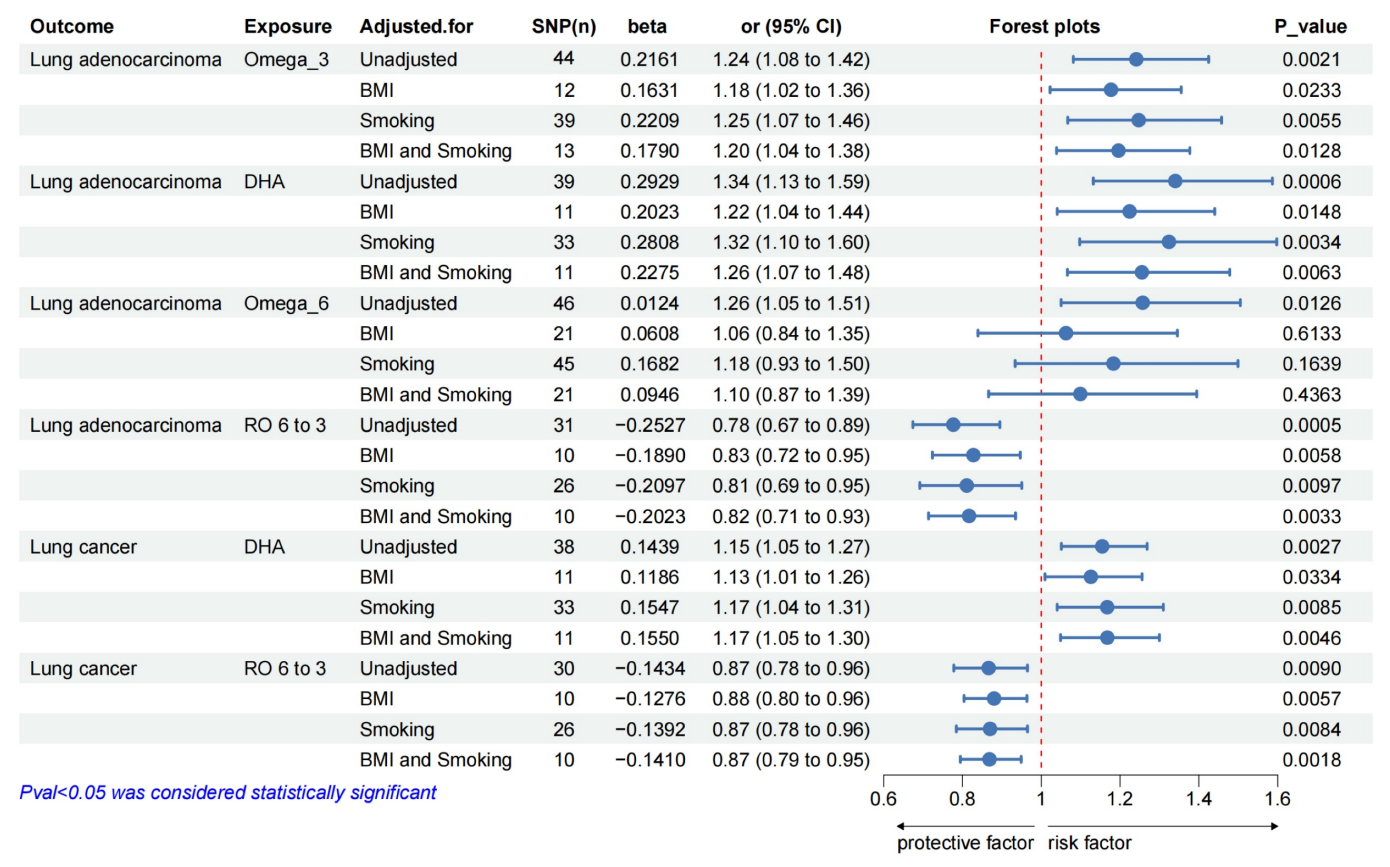
Multivariate MR effect estimates of PUFAs on overall lung cancer and lung adenocarcinoma, adjusted for BMI and Smoking. Abbreviation: MR = Mendelian randomization, Omega_3 =Omega_3 acid, DHA = Docosahexaenoic acid, Omega_6 = Omega_6 acid, RO 6 to 3 = Ratio of omega-6 fatty acids to omega-3 fatty acids.

Univariate MR analyses showed that higher genetically predicted levels of DHA and omega-6/omega-3 were associated with overall lung cancer, while omega-3, DHA, omega-6 and omega-6/omega-3 were associated with the risk of LUAD. So, we estimated the independent effects of circulating PUFAs (omega-3, DHA, omega-6 and omega-6/omega-3) on overall lung cancer and LUAD using multivariable MR conditioned on BMI and smoking (including current tobacco smoking, smoking/smokers in household and past tobacco smoking) (Figure 2). More details of genetic instrument source for BMI and smoking traits in Supplementary Table S9 and MVMR results in Supplementary Table S10-13. And we performed MR analyses in the reverse direction to estimate the causal effects of overall lung cancer and LUAD on the PUFAs (omega-3, DHA, omega-6 and omega-6/omega-3) separately, which could also help determine the correct direction of causality. Sensitivity analyses of the raw bidirectional MR analysis and the adjusted MR analysis in the Supplementary Table S14.

#### 3.3.1. Results of Multivariable MR

Using multivariate MR, we also found that circulating levels of omega-3, DHA and omega-6/omega-3 affected overall risk of lung cancer or LUAD, independent of BMI and smoking (Figure 2). After adjusting for BMI, omega-3 (LUAD: OR_MVMR: 1.18; 95% CI: 1.02 to 1.36; *P*_value = 0.0233) or DHA (overall lung cancer: OR_MVMR: 1.13; 95% CI: 1.01 to 1.26; *P*_value = 0.0334; LUAD: OR_MVMR: 1.22; 95% CI: 1.04 to 1.44; *P*_value = 0.0148) was associated with an increased overall risk of lung cancer or LUAD (Figure 2), while omega-6/omega-3 (overall lung cancer: OR_MVMR: 0.88; 95% CI: 0.80 to 0.96; *P*_value = 0.0057; LUAD: OR_MVMR: 0.83; 95% CI: 0.72 to 0.95; *P*_value = 0.0058) was associated with a decreased overall risk of lung cancer and LUAD (Figure 2).

After adjustment of smoking, there was a genetic predictive relationship between omega-3 (LUAD: OR_MVMR: 1.25; 95% CI: 1.07 to 1.46; *P*_value = 0.0055) or DHA (overall lung cancer: OR_MVMR: 1.17; 95% CI: 1.04 to 1.31; *P*_value = 0.0085; LUAD: OR_MVMR: 1.32; 95% CI: 1.10 to 1.60; *P*_value = 0.0034) and overall risk of lung cancer or LUAD (Figure 2), and omega-6/omega-3 (overall lung cancer: OR_MVMR: 0.87; 95% CI: 0.78 to 0.96; *P*_value = 0.0084; LUAD: OR_MVMR: 0.81; 95% CI: 0.69 to 0.95; *P*_value = 0.0097) was associated with a low risk of overall lung cancer and LUAD (Figure 2).

When adjusting for both of BMI and smoking, there was a genetic association between omega-3 (LUAD: OR_MVMR: 1.20; 95% CI: 1.04 to 1.38; *P*_value = 0.0128) or DHA (overall lung cancer: OR_MVMR: 1.17; 95% CI: 1.05 to 1.30; *P*_value = 0.0046; LUAD: OR_MVMR: 1.26; 95% CI: 1.07 to 1.48; *P*_value = 0.0063) and overall lung cancer or LUAD (Figure 2). Similarly, genetic correlations between omega-6/omega-3 (overall lung cancer: OR_MVMR: 0.87; 95% CI: 0.79 to 0.95; *P*_value = 0.0018; LUAD: OR_MVMR: 0.82; 95% CI: 0.71 to 0.93; *P*_value = 0.0033) and overall lung cancer or LUAD also were found (Figure 2).

However, no significant association was observed between omega-6 levels and overall lung cancer or LUAD after adusting for BMI and smoking.

#### 3.3.1. Resulits of Bidirectional MR analyses

**Figure 3:**
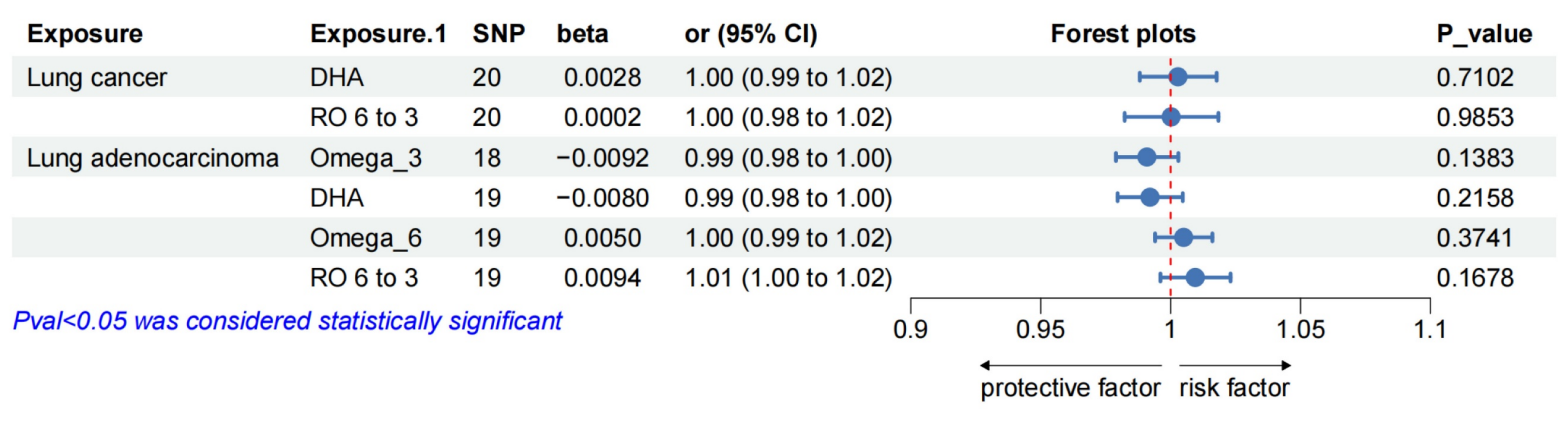
Reciprocal MR effect estimates of overall lung cancer and lung adenocarcinoma on PUFAs. Abbreviation: MR = Mendelian randomization, Omega_3 =Omega_3 acid, DHA = Docosahexaenoic acid, Omega_6 = Omega_6 acid, RO 6 to 3 = Ratio of omega-6 fatty acids to omega-3 fatty acids.

Furthermore, we estimated the effects of overall lung cancer on omega-6, DHA, omega-6 and omega-6/omega-3, and LUAD on DHA and omega-6/omega-3. No heterogeneity or directional pleiotropy was found in the reverse direction MR analyses after removal of all outliers (all details in the Supplementary Table S14). No significant genetic association was observed between PUFAs (omega-6, DHA, omega-6 and omega-6/omega-3) and lung cancers (over lung cancer and LUAD), in the results of reverse MR analyses (Figure 3).

A forest plot of the causal estimates of PUFAs on the risk of lung cancer is shown in Figure 2. Overall, the consistency of effect sizes of different methods indicates that confidence in the results of each analysis. The corresponding scatter plots for the two-sample MR analyzes with positive results (*P*_value < 0.05) are shown in Supplementary Figures S2-7. And the leave-one-out stability tests conducted by excluding a single SNP at a time are described in detail in the Supplementary Figures S8-13.

## 4. Discussion

In this study, we examined the link between PUFA and lung cancer risk using summarized data from a comprehensive GWAS. Our focus was on the associations of two primary PUFA families, omega-3 and omega-6, including their major subtypes, with overall lung cancer, as well as two significant histological subtypes, LUAD and SQLC. The primary isoforms of omega-3 studied were DHA and EPA, while for omega-6, they were LA and AA. Our findings indicated no significant association between omega-3 (DHA, EPA) and omega-6 (LA, AA) with the risk of SQLC. However, omega-3 and DHA were positively linked with LUAD risk, and DHA was also associated with an increased overall lung cancer risk. The omega-6 family showed a positive association with LUAD risk, but this correlation was not evident after adjusting for BMI and smoking effects. Notably, the ratio of omega-6 to omega-3 demonstrated a negative correlation with the risk of both overall lung cancer and LUAD.

Previous observational studies examining the relationship between specific types of PUFA and lung cancer risk have yielded inconclusive results, leading to controversy over the clinical significance of omega-3 and omega-6. Chen et al. reported a negative association between lung cancer risk and the intake of omega-3 (hazard ratio [HR], 0.82; 95%; CI, 0.73-0.93 per 1 g/day) and omega-6 (HR, 0.98; 95% CI, 0.96-0.99 per 1 g/day) 61. Conversely, Luu et al. found that total PUFA intake was inversely correlated with lung cancer risk, with hazard ratios and 95% confidence intervals of 0.84 (0.71-0.98), 0.97 (0.83-1.13), 0.86 (0.74-1.01), and 0.85 (0.73-1.00) for quintiles 2 through 5 compared with quintile 1, respectively (*P* trend = 0.11). Notably, DHA and EPA intake showed a positive correlation with lung cancer risk, whereas the omega-6/omega-3 ratio was inversely associated with lung cancer risk, particularly among non-smokers and LUAD patients 62. While a recent systematic assessment and meta-analysis showed that consumption of fish rich in DHA and EPA was associated with a reduced risk of lung cancer 63. Meanwhile, another meta-analysis based on eight prospective cohort studies showed no significant effect of polyunsaturated fatty acid intake on lung cancer risk 17. Observational studies can be prone to unmeasured confounding or reverse causality, resulting in inconsistent findings. Therefore, we employed MR analysis to investigate the causal effects of specific PUFA subtypes on lung cancer risk. Our findings indicated that EPA, LA, and AA were not significantly associated with overall lung cancer, LUAD, or SQLC. However, DHA was positively associated with the risk of both overall lung cancer and LUAD, corroborating the results of a recent MR study on DHA and lung cancer risk 64. Prior research has highlighted DHA's unique role in tumorigenesis, including its propensity to promote oxidation at high doses and influence cell permeability, motility, and signaling 65. Moreover, dietary intake of DHA significantly increased DHA levels in lung tissues 66. Oxidative stress is one of the molecular mechanisms by which DHA acts, and increased production of oxidative stress markers may be one of the reasons why DHA increases the risk of lung cancer 67. Our findings support this, providing evidence that DHA heightens the risk of lung cancer. From a clinical perspective, these results suggest that dietary intake of omega-3 and DHA should be carefully moderated. For instance, while fatty fish (e.g., salmon, mackerel) and plant-based sources (e.g., flaxseeds, walnuts) are recommended for their cardioprotective benefits, excessive consumption may paradoxically elevate LUAD risk. Similarly, omega-3 supplements such as fish oil capsules or algae-based formulations should be used judiciously, particularly in high-risk populations. Public health guidelines could emphasize balancing omega-6 and omega-3 intake to achieve a higher omega-6/omega-3 ratio, which our study identifies as protective. This approach aligns with existing recommendations for cancer prevention while addressing the potential risks of overconsumption.

Another intriguing finding in our study was that omega-3, omega-6, and DHA were positively correlated with LUAD but showed no clinical significance for SQLC. This discrepancy may be linked to the distinct pathological types of lung cancer. Adenocarcinoma is more prevalent in women, and estrogen plays a crucial role in the development and progression of LUAD. Studies have shown that estrogen levels in peripheral lung tissue of patients with synchronous multiple LUADs are significantly higher compared to control subjects 68. Additionally, estrogen receptor gene SNPs have been associated with an increased risk of LUAD 69. Numerous animal and clinical studies have also demonstrated that estrogen stimulation enhances the biosynthesis of omega-3, omega-6, and DHA 70-73. Therefore, the complex interaction between omega-3, omega-6, DHA, and estrogen could explain our findings.

This study is the first to employ multivariable and bidirectional Mendelian randomization to investigate the causal role of the omega-6/omega-3 ratio in lung cancer. Unlike previous MR studies that focused on isolated PUFA subtypes (e.g., DHA, EPA), our comprehensive analysis of seven PUFA traits, including the ratio, reveals a unique protective effect against LUAD. This finding aligns with observational studies suggesting that lipid balance modulates cancer progression but extends their conclusions by establishing causality. By adjusting for BMI and smoking through multivariable MR, we address key confounders that biased prior observational studies. Furthermore, bidirectional MR analyses confirmed that reverse causality (e.g., lung cancer altering PUFA metabolism) is unlikely, strengthening the robustness of our conclusions. These methodological choices distinguish our work from earlier MR investigations 64, 74 and provide a framework for future studies on dietary factors in cancer. Previous studies have highlighted that characteristic lipid profiles associated with tumor growth and progression can drive changes in the growth characteristics of cancerous lesions. Alterations in the omega-6 to omega-3 ratio can modify the lipid profile and cell membrane fatty acid content of cancer cells, impacting oxidative status, inflammation, and cellular signaling. These changes disrupt the protective environment of cancer cells, promoting a shift from proliferative survival to cell removal and/or cell death 18. Two recent population-based cohort studies, SWHS and SMHS, have shown that the ratio of omega-6/omega-3 (i.e., 7:1) is negatively correlated with the risk of lung cancer 62. Additionally, findings from a prospective follow-up of participants in the Chinese Health and Nutrition Survey (CHNS, n = 14,117) and the National Health and Nutrition Examination Survey (NHANES, n = 36,032) showed that PUFA intake at an omega-6/omega-3 ratio of 6-10 was associated with a lower risk of mortality 75. Despite our analysis confirming the negative association between the omega-6/omega-3 ratio and lung cancer risk, the number of studies examining this ratio in relation to lung cancer risk is limited. Therefore, replication of this study in broader populations is necessary.

BMI and smoking are confounding factors when evaluating the relationship between PUFA and lung cancer. To address this, we used multivariate Mendelian Randomization (MR) to eliminate the biases caused by BMI and smoking. The results indicated that omega-3 and DHA remained risk factors for lung cancer and LUAD, while the omega-6/omega-3 ratio continued to be a protective factor for both. However, after adjusting for BMI and smoking, no causal relationship between omega-6 and lung cancer risk was observed. Previous research has shown that SNPs associated with BMI and smoking have a causal effect on overall lung cancer in smokers, suggesting potential interactions between omega-6, BMI, and smoking in lung cancer development. These findings imply that smoking and BMI might be mediators in the relationship between omega-6 and lung cancer risk. Further analyses, such as network Mendelian Randomization, which utilizes genetic instrumentation to study mediators in causal pathways, may offer deeper insights into these interactions 76. Our analysis highlights the significant role of omega-3 and its subtype DHA in lung cancer risk. Omega-3 is widely used as an immune nutrient in the nutritional treatment of cancer due to its crucial roles in cell signaling, structure, and membrane fluidity 77-79. Additionally, omega-3 mediates inflammation reduction, exerting anti-inflammatory effects 80-82. However, a meta-analysis found no significant correlation between omega-3 fatty acid intake and lung cancer risk 83. Retrospective studies also indicated that although omega-3 fatty acids reduced C-reactive protein and IL-6 levels in patients with advanced non-small cell lung cancer (NSCLC), they did not affect nutritional status or quality of life 84. Our MR analysis showed a positive correlation between omega-3 levels and lung cancer risk, suggesting that moderate control of omega-3 and DHA intake may be crucial for lung cancer prevention.

Our analysis presents several significant strengths. First, we conducted separate subgroup analyses of PUFA and lung cancer, making this one of the most comprehensive studies to characterize the correlation between PUFA and lung cancer. Second, employing two-sample, multivariate MR, and bidirectional MR analyses mitigates bias from confounding factors and reverse causality, allowing sensitivity analyses to identify and adjust for pleiotropy.

However, this study has several limitations and future directions:

European-Centric Data: Our findings are derived from European ancestry cohorts, which may limit generalizability to other populations with distinct genetic backgrounds, dietary habits, or environmental exposures. For example, Asian populations typically consume higher omega-3 levels from seafood, potentially altering the omega-6/omega-3 ratio's protective effects. Future replication in diverse cohorts (e.g., African, East Asian) is critical to validate our results.

Need for Experimental Validation: While MR provides robust causal inference, mechanistic validation is essential. We propose three future directions:

- In vitro studies: Treating lung adenocarcinoma cell lines (e.g., A549, H1299) with varying omega-6/omega-3 ratios to assess proliferation, apoptosis, and inflammatory markers (e.g., COX-2, IL-6).
- Animal models: Testing high vs. low omega-6/omega-3 diets in carcinogen-induced or genetically engineered lung cancer mice.
- Human trials: Conducting randomized controlled trials (RCTs) to evaluate dietary interventions targeting the omega-6/omega-3 ratio in high-risk populations.
- Integration with Omics Data: Combining MR with metabolomics or lipidomics profiling in lung cancer tissues could uncover biomarkers linking PUFA ratios to tumor progression.

## 5. Conclusions

This Mendelian randomization study underscores the dual role of PUFAs in lung cancer pathogenesis. Using multivariable and bidirectional MR analyses—a methodological advance over prior studies—we provide the first causal evidence that a higher omega-6/omega-3 ratio significantly reduces both overall lung cancer (OR = 0.87) and LUAD risk (OR = 0.78), independent of BMI and smoking. Conversely, genetically predicted omega-3 and DHA levels were associated with elevated LUAD risk. These findings highlight the ratio's unique potential to modulate inflammatory pathways and lipid metabolism, offering a novel dietary strategy for lung cancer prevention. Our study redefines the role of PUFA balance in lung cancer prevention and calls for a paradigm shift from isolated nutrient-focused guidelines to holistic dietary patterns that prioritize omega-6/omega-3 equilibrium.

## Supplementary Material

Supplementary materials and figures.

Supplementary tables.

## Figures and Tables

**Table 1:** All IVW results of univariable Mendelian randomization analyses exploring associations between PUFAs and risk of lung cancers.

Exposure	Outcome	Method	SNP	beta	*P*_value	OR (95% CI)
Omega-3	Lung cancer	IVW-random	42	0.0048	0.9469	1.00 (0.87 to 1.16)
	Lung adenocarcinoma	IVW	44	0.2161	0.0021	1.24 (1.08 to 1.42)
	Squamous cell lung cancer	IVW	41	-0.0876	0.3791	0.92 (0.75 to 1.11)
DHA	Lung cancer	IVW-random	38	0.1439	0.0027	1.15 (1.05 to 1.27)
	Lung adenocarcinoma	IVW	39	0.1439	0.0027	1.34 (1.13 to 1.59)
	Squamous cell lung cancer	IVW	36	-0.1430	0.2205	0.87 (0.69 to 1.09)
EPA	Lung cancer	IVW	5	0.1946	0.6748	1.21 (0.49 to 3.02)
	Lung adenocarcinoma	IVW	7	1.0541	0.0621	2.87 (0.95 to 8.68)
	Squamous cell lung cancer	IVW	6	-0.0986	0.8810	0.91 (0.25 to 3.29)
Omega-6	Lung cancer	IVW	48	0.0124	0.8463	1.01 (0.89 to 1.15)
	Lung adenocarcinoma	IVW	46	0.2291	0.0126	1.26 (1.05 to 1.51)
	Squamous cell lung cancer	IVW	49	-0.0497	0.6288	0.95 (0.78 to 1.16)
LA	Lung cancer	IVW	42	-0.0002	0.9974	1.00 (0.88 to 1.14)
	Lung adenocarcinoma	IVW	41	0.1668	0.1070	1.18 (0.96 to 1.45)
	Squamous cell lung cancer	IVW	43	-0.0872	0.4093	0.92 (0.74 to 1.13)
AA	Lung cancer	IVW	13	0.7366	0.0557	2.09 (0.98 to 4.44)
	Lung adenocarcinoma	IVW	13	0.9326	0.0783	2.54 (0.90 to 7.18)
	Squamous cell lung cancer	IVW-random	13	0.8002	0.2093	2.23 (0.64 to 7.76)
omega-6/omega-3 ratio	Lung cancer	IVW-random	30	-0.1434	0.0090	0.87 (0.78 to 0.96)
	Lung adenocarcinoma	IVW	31	-0.2527	0.0005	0.78 (0.67 to 0.89)
	Squamous cell lung cancer	IVW	28	0.0953	0.4653	1.10 (0.85 to 1.42)

OR: odds ratio; CIs: confidence intervals; IVW-random: random effect. The total results using for the MR analysis using different Mendelian randomization statistical models are shown in Supplementary Table S8.

**Table 2:** Sensitivity analyses of the raw MR analysis and the adjusted MR analysis (adjusted by excluding all outliers and heterogeneous SNPs identified by the MR-PRESSO).

Exposure	Outcome	nIVs	Heterogeneity test		MR-Egger pleiotropy test		MR-PRESSO global test		MR-PRESSO distorted outlier test		Heterogeneity test adjusted	
			MREgger Q (*P*-value)	IVW Q (*P*-value)	Intercept (*P*-value)	Intercept adjusted *(P*-value)	RSSobs (*P*-value)	Adjusted RSSobs (*P*-value)	Outlying SNPs (*P-*value<0.05)	Outlying SNPs *(P*-value<1)	MR Egger adjusted Q (*P*-value)	IVW adjusted Q (*P-*value)
Omega-3	LC	42	82.5417 (0.0002)	85.6823 (0.0001)	-0.0085 (0.2132)	0.0014 (0.8562)	98.5192 (0.0041)	58.6513 (0.0615)	rs174564, rs2394976	None	56.1368 (0.0466)	56.1835 (0.0574)
	LUAD	44	57.9650 (0.0514)	58.4754 (0.0579)	-0.0055 (0.5463)	NA	61.0428 (0.0974)	NA	None	None	NA	NA
	SCLC	41	77.4377 (0.0007)	82.3404 (0.0003)	-0.0161 (0.1104)	-0.0041 (0.6963)	100.6726 (0.0077)	46.1960 (0.3084)	rs139974673, rs174564, rs2394976	None	43.8690 (0.2727)	44.0428 (0.3045)
DHA	LC	38	56.9144 (0.0192)	60.2939 (0.0121)	-0.0096 (0.1467)	-0.0096 (0.1467)	69.4713 (0.0425)	42.3353 (0.4894)	rs2394976	None	33.7857 (0.5743)	35.5243 (0.5383)
	LUAD	39	53.9483 (0.0355)	54.05 (0.0440)	-0.0025 (0.7930)	NA	54.9875 (0.0829)	NA	None	None	NA	NA
	SCLC	36	56.4246 (0.0213)	62.2976 (0.0077)	-0.0194 (0.0572)	0.0003 (0.9778)	82.7734 (0.0421)	24.409 (0.9424)	rs139974673, rs174564, rs2394976	None	23.3760 (0.9148)	23.3768 (0.9332)
EPA	LC	5	4.4315 (0.4891)	11.0820 (0.0859)	-0.0646 (0.0495)	-0.0352 (0.3203)	21.1815 (0.1106)	4.3771 (0.7113)	None	rs174556, rs179976	0.4781 (0.9237)	1.8897 (0.7560)
	LUAD	7	7.9297 (0.1602)	8.1801 (0.2252)	-0.0182 (0.7075)	NA	16.0993 (0.2039)	NA	None	None	NA	NA
	SCLC	6	3.3970 (0.6390)	10.0124 (0.1241)	-0.1011 (0.0499)	-0.0701 (0.1932)	18.6169 (0.1607)	4.9786 (0.6425)	None	rs174556	1.3277 (0.8566)	3.7689 (0.5831)
Omega-6	LC	48	91.9865 (9.623e-05)	94.3291 (7.46e-05)	-0.0102 (0.2795)	-0.0124 (0.0942)	106.5887 (<1e-04)	66.6582 (0.0522)	rs28383314, rs79429216	None	51.9276 (0.2220)	55.3002 (0.1637)
	LUAD	46	76.5984 (0.0041)	76.8722 (0.0051)	-0.0053 (0.6838)	-0.0139 (0.1885)	84.7636 (0.0022)	46.6748 (0.4949)	rs28383314	rs1002687, rs5754102, rs79429216	38.3991 (0.6709)	40.1843 (0.6359)
	SCLC	49	73.6822 (0.0077)	75.4010 (0.0070)	-0.0134 (0.3004)	-0.0192 (0.1087)	81.1957 (0.0058)	67.9294 (0.0535)	rs79429216	None	59.0915 (0.0932)	62.5293 (0.0642)
LA	LC	42	77.9955 (0.0003)	78.1413 (0.0004)	-0.0029 (0.7859)	-0.0122 (0.1316)	92.7097 (<1e-04)	54.6853 (0.1169)	rs174564, rs79429216	None	39.8469 (0.3879)	42.3366 (0.3290)
	LUAD	41	62.6224 (0.0126)	62.6234 (0.0164)	-0.0004 (0.9796)	-0.0092 (0.4638)	74.7348 (0.0045)	51.0702 (0.1708)	rs174564	rs1002687, rs7816447	41.1830 (0.2925)	41.7931 (0.3095)
	SCLC	43	63.9807 (0.0094)	64.1167 (0.0120)	-0.0043 (0.7721)	-0.0110 (0.4181)	70.5631 (0.0113)	57.7268 (0.0954)	rs79429216	None	50.7865 (0.0979)	51.6584 (0.1024)
AA	LC	11	14.9306 (0.0929)	16.9113 (0.0763)	-0.0144 (0.3029)	NA	37.6304 (0.1404)	NA	None	None	NA	NA
	LUAD	11	10.5666 (0.3066)	14.1731 (0.1652)	-0.0297 (0.1136)	NA	35.613 (0.1713)	NA	None	None	NA	NA
	SCLC	11	20.3167 (0.0161)	20.4058 (0.0256)	-0.0047 (0.8470)	NA	37.3593 (0.1305)	NA	None	None	NA	NA
omega-6/omega-3 ratio	LC	30	72.4508 (4.980e-06)	72.4783 (8.323e-06)	0.0009 (0.9201)	-0.0025 (0.7321)	77.5993 (0.0097)	50.6532 (0.0814)	rs2394976	NA	47.4975 (0.0061)	47.7162 (0.0082)
	LUAD	31	37.7891 (0.081)	38.0868 (0.0968)	-0.0045 (0.6484)	NA	39.4101 (0.2586)	NA	None	NA	NA	NA
	SCLC	28	59.4216 (3.139e-04)	64.8614 (9.405e-05)	0.0195 (0.1276)	0.0051 (0.7260)	95.1956 (0.0491)	29.5675 (0.4428)	rs139974673, rs174564, rs2394976	NA	27.4380 (0.2845)	27.5817 (0.3275)

As the Supplementary Figure 1 showed, for the process of adjustment, we firstly did a raw MR analysis and got an uncorrected causal evaluation. Then, MR-PRESSO global and Outliers test was performed to find unstable SNPs, and an adjusted MR analysis was performed again after removing all unstable SNPs, and the heterogeneity, pleiotropy and causal effect values were re-evaluated. MR: Mendelian randomization analysis; LC = Lung Cancer; LUAD = Lung adenocarcinoma; SCLC = Squamous cell lung cancer; nIVs: Number of instrumental variables; Omega_3 =Omega_3 acid, DHA = Docosahexaenoic acid, EPA = Eicosapentaenoate acid, Omega_6 = Omega_6 acid, LA = Linoleic acid, AA = Arachidonic acid.

## References

[1] Forman BM, Chen J, Evans RM (1997). Hypolipidemic drugs, polyunsaturated fatty acids, and eicosanoids are ligands for peroxisome proliferator-activated receptors alpha and delta. Proc Natl Acad Sci U S A.

[2] Dyall SC, Balas L, Bazan NG, Brenna JT, Chiang N, da Costa Souza F, Dalli J, Durand T, Galano JM, Lein PJ (2022). Polyunsaturated fatty acids and fatty acid-derived lipid mediators: Recent advances in the understanding of their biosynthesis, structures, and functions. Prog Lipid Res.

[3] Massey KA, Nicolaou A (2013). Lipidomics of oxidized polyunsaturated fatty acids. Free Radic Biol Med.

[4] Chen SC, Chen PY, Wu YL, Chen CW, Chen HW, Lii CK, Sun HL, Liu KL (2016). Long-chain polyunsaturated fatty acids amend palmitate-induced inflammation and insulin resistance in mouse C2C12 myotubes. Food Funct.

[5] Borsini A, Nicolaou A, Camacho-Muñoz D, Kendall AC, Di Benedetto MG, Giacobbe J, Su KP, Pariante CM (2021). Omega-3 polyunsaturated fatty acids protect against inflammation through production of LOX and CYP450 lipid mediators: relevance for major depression and for human hippocampal neurogenesis. Mol Psychiatry.

[6] Cao W, Wang C, Chin Y, Chen X, Gao Y, Yuan S, Xue C, Wang Y, Tang Q (2019). DHA-phospholipids (DHA-PL) and EPA-phospholipids (EPA-PL) prevent intestinal dysfunction induced by chronic stress. Food Funct.

[7] Brouwers H, Jónasdóttir HS, Kuipers ME, Kwekkeboom JC, Auger JL, Gonzalez-Torres M, López-Vicario C, Clària J, Freysdottir J, Hardardottir I (2020). Anti-Inflammatory and Proresolving Effects of the Omega-6 Polyunsaturated Fatty Acid Adrenic Acid. J Immunol.

[8] Schwärzler J, Mayr L, Vich Vila A, Grabherr F, Niederreiter L, Philipp M, Grander C, Meyer M, Jukic A, Tröger S (2022). PUFA-Induced Metabolic Enteritis as a Fuel for Crohn's Disease. Gastroenterology.

[9] Vila IK, Chamma H, Steer A, Saccas M, Taffoni C, Turtoi E, Reinert LS, Hussain S, Marines J, Jin L (2022). STING orchestrates the crosstalk between polyunsaturated fatty acid metabolism and inflammatory responses. Cell Metab.

[10] Dierge E, Debock E, Guilbaud C, Corbet C, Mignolet E, Mignard L, Bastien E, Dessy C, Larondelle Y, Feron O (2021). Peroxidation of n-3 and n-6 polyunsaturated fatty acids in the acidic tumor environment leads to ferroptosis-mediated anticancer effects. Cell Metab.

[11] Hidaka A, Shimazu T, Sawada N, Yamaji T, Iwasaki M, Sasazuki S, Inoue M, Tsugane S (2015). Fish, n-3 PUFA consumption, and pancreatic cancer risk in Japanese: a large, population-based, prospective cohort study. Am J Clin Nutr.

[12] Ji XW, Wang J, Shen QM, Li ZY, Jiang YF, Liu DK, Tan YT, Li HL, Xiang YB (2021). Dietary fat intake and liver cancer incidence: A population-based cohort study in Chinese men. Int J Cancer.

[13] Serini S, Calviello G (2017). Modulation of Ras/ERK and Phosphoinositide Signaling by Long-Chain n-3 PUFA in Breast Cancer and Their Potential Complementary Role in Combination with Targeted Drugs. Nutrients.

[14] Sung H, Ferlay J, Siegel RL, Laversanne M, Soerjomataram I, Jemal A, Bray F (2021). Global Cancer Statistics 2020: GLOBOCAN Estimates of Incidence and Mortality Worldwide for 36 Cancers in 185 Countries. CA Cancer J Clin.

[15] Bade BC, Dela Cruz CS (2020). Lung Cancer 2020: Epidemiology, Etiology, and Prevention. Clin Chest Med.

[16] Wei X, Zhu C, Ji M, Fan J, Xie J, Huang Y, Jiang X, Xu J, Yin R, Du L (2021). Diet and Risk of Incident Lung Cancer: A Large Prospective Cohort Study in UK Biobank. Am J Clin Nutr.

[17] Zhang YF, Lu J, Yu FF, Gao HF, Zhou YH (2014). Polyunsaturated fatty acid intake and risk of lung cancer: a meta-analysis of prospective studies. PLoS One.

[18] Abel S, Riedel S, Gelderblom WC (2014). Dietary PUFA and cancer. Proc Nutr Soc.

[19] Bai X, Shao J, Zhou S, Zhao Z, Li F, Xiang R, Zhao AZ, Pan J (2019). Inhibition of lung cancer growth and metastasis by DHA and its metabolite, RvD1, through miR-138-5p/FOXC1 pathway. J Exp Clin Cancer Res.

[20] Zhang G, Panigrahy D, Mahakian LM, Yang J, Liu JY, Stephen Lee KS, Wettersten HI, Ulu A, Hu X, Tam S (2013). Epoxy metabolites of docosahexaenoic acid (DHA) inhibit angiogenesis, tumor growth, and metastasis. Proc Natl Acad Sci U S A.

[21] Yang P, Cartwright C, Chan D, Ding J, Felix E, Pan Y, Pang J, Rhea P, Block K, Fischer SM, Newman RA (2014). Anticancer activity of fish oils against human lung cancer is associated with changes in formation of PGE2 and PGE3 and alteration of Akt phosphorylation. Mol Carcinog.

[22] Montecillo-Aguado M, Tirado-Rodriguez B, Antonio-Andres G, Morales-Martinez M, Tong Z, Yang J, Hammock BD, Hernandez-Pando R, Huerta-Yepez S (2022). Omega-6 Polyunsaturated Fatty Acids Enhance Tumor Aggressiveness in Experimental Lung Cancer Model: Important Role of Oxylipins. Int J Mol Sci.

[23] Yang JJ, Yu D, Takata Y, Smith-Warner SA, Blot W, White E, Robien K, Park Y, Xiang YB, Sinha R (2017). Dietary Fat Intake and Lung Cancer Risk: A Pooled Analysis. J Clin Oncol.

[24] Sekula P, Del Greco MF, Pattaro C, Köttgen A (2016). Mendelian Randomization as an Approach to Assess Causality Using Observational Data. J Am Soc Nephrol.

[25] Holmes MV, Ala-Korpela M, Smith GD (2017). Mendelian randomization in cardiometabolic disease: challenges in evaluating causality. Nat Rev Cardiol.

[26] Smith GD, Ebrahim S (2004). Mendelian randomization: prospects, potentials, and limitations. Int J Epidemiol.

[27] Emdin CA, Khera AV, Kathiresan S (2017). Mendelian Randomization. Jama.

[28] Burgess S, Foley CN, Allara E, Staley JR, Howson JMM (2020). A robust and efficient method for Mendelian randomization with hundreds of genetic variants. Nat Commun.

[29] Skrivankova VW, Richmond RC, Woolf BAR, Yarmolinsky J, Davies NM, Swanson SA, VanderWeele TJ, Higgins JPT, Timpson NJ, Dimou N (2021). Strengthening the Reporting of Observational Studies in Epidemiology Using Mendelian Randomization: The STROBE-MR Statement. Jama.

[30] Davies NM, Holmes MV, Davey Smith G (2018). Reading Mendelian randomisation studies: a guide, glossary, and checklist for clinicians. Bmj.

[31] Davey Smith G, Hemani G (2014). Mendelian randomization: genetic anchors for causal inference in epidemiological studies. Hum Mol Genet.

[32] Smith GD, Ebrahim S (2003). 'Mendelian randomization': can genetic epidemiology contribute to understanding environmental determinants of disease?. Int J Epidemiol.

[33] Bowden J, Del Greco MF, Minelli C, Davey Smith G, Sheehan NA, Thompson JR (2016). Assessing the suitability of summary data for two-sample Mendelian randomization analyses using MR-Egger regression: the role of the I2 statistic. Int J Epidemiol.

[34] Sanderson E, Windmeijer F (2016). A weak instrument [Formula: see text]-test in linear IV models with multiple endogenous variables. J Econom.

[35] Manousaki D, Harroud A, Mitchell RE, Ross S, Forgetta V, Timpson NJ, Smith GD, Polychronakos C, Richards JB (2021). Vitamin D levels and risk of type 1 diabetes: A Mendelian randomization study. PLoS Med.

[36] Julkunen H, Cichońska A, Slagboom PE, Würtz P (2021). Metabolic biomarker profiling for identification of susceptibility to severe pneumonia and COVID-19 in the general population. Elife.

[37] Soininen P, Kangas AJ, Würtz P, Tukiainen T, Tynkkynen T, Laatikainen R, Järvelin MR, Kähönen M, Lehtimäki T, Viikari J (2009). High-throughput serum NMR metabonomics for cost-effective holistic studies on systemic metabolism. Analyst.

[38] Würtz P, Kangas AJ, Soininen P, Lawlor DA, Davey Smith G, Ala-Korpela M (2017). Quantitative Serum Nuclear Magnetic Resonance Metabolomics in Large-Scale Epidemiology: A Primer on -Omic Technologies. Am J Epidemiol.

[39] Shin SY, Fauman EB, Petersen AK, Krumsiek J, Santos R, Huang J, Arnold M, Erte I, Forgetta V, Yang TP (2014). An atlas of genetic influences on human blood metabolites. Nat Genet.

[40] Krumsiek J, Suhre K, Evans AM, Mitchell MW, Mohney RP, Milburn MV, Wägele B, Römisch-Margl W, Illig T, Adamski J (2012). Mining the unknown: a systems approach to metabolite identification combining genetic and metabolic information. PLoS Genet.

[41] Suhre K, Shin SY, Petersen AK, Mohney RP, Meredith D, Wägele B, Altmaier E, Deloukas P, Erdmann J, Grundberg E (2011). Human metabolic individuality in biomedical and pharmaceutical research. Nature.

[42] Pesatori AC, Carugno M, Consonni D, Hung RJ, Papadoupolos A, Landi MT, Brenner H, Müller H, Harris CC, Duell EJ (2013). Hormone use and risk for lung cancer: a pooled analysis from the International Lung Cancer Consortium (ILCCO). Br J Cancer.

[43] Wang Y, McKay JD, Rafnar T, Wang Z, Timofeeva MN, Broderick P, Zong X, Laplana M, Wei Y, Han Y (2014). Rare variants of large effect in BRCA2 and CHEK2 affect risk of lung cancer. Nat Genet.

[44] Burgess S, Bowden J, Fall T, Ingelsson E, Thompson SG (2017). Sensitivity Analyses for Robust Causal Inference from Mendelian Randomization Analyses with Multiple Genetic Variants. Epidemiology.

[45] Bowden J, Davey Smith G, Burgess S (2015). Mendelian randomization with invalid instruments: effect estimation and bias detection through Egger regression. Int J Epidemiol.

[46] Bowden J, Davey Smith G, Haycock PC, Burgess S (2016). Consistent Estimation in Mendelian Randomization with Some Invalid Instruments Using a Weighted Median Estimator. Genet Epidemiol.

[47] Hartwig FP, Davey Smith G, Bowden J (2017). Robust inference in summary data Mendelian randomization via the zero modal pleiotropy assumption. Int J Epidemiol.

[48] Bowden J, Del Greco MF, Minelli C, Zhao Q, Lawlor DA, Sheehan NA, Thompson J, Davey Smith G (2019). Improving the accuracy of two-sample summary-data Mendelian randomization: moving beyond the NOME assumption. Int J Epidemiol.

[49] Bowden J, Del Greco MF, Minelli C, Davey Smith G, Sheehan N, Thompson J (2017). A framework for the investigation of pleiotropy in two-sample summary data Mendelian randomization. Stat Med.

[50] Verbanck M, Chen CY, Neale B, Do R (2018). Detection of widespread horizontal pleiotropy in causal relationships inferred from Mendelian randomization between complex traits and diseases. Nat Genet.

[51] Greco MF, Minelli C, Sheehan NA, Thompson JR (2015). Detecting pleiotropy in Mendelian randomisation studies with summary data and a continuous outcome. Stat Med.

[52] Burgess S, Thompson SG (2017). Interpreting findings from Mendelian randomization using the MR-Egger method. Eur J Epidemiol.

[53] Slob EAW, Burgess S (2020). A comparison of robust Mendelian randomization methods using summary data. Genet Epidemiol.

[54] Burgess S, Thompson SG (2015). Multivariable Mendelian randomization: the use of pleiotropic genetic variants to estimate causal effects. Am J Epidemiol.

[55] Sanderson E, Davey Smith G, Windmeijer F, Bowden J (2019). An examination of multivariable Mendelian randomization in the single-sample and two-sample summary data settings. Int J Epidemiol.

[56] Yengo L, Sidorenko J, Kemper KE, Zheng Z, Wood AR, Weedon MN, Frayling TM, Hirschhorn J, Yang J, Visscher PM (2018). Meta-analysis of genome-wide association studies for height and body mass index in ∼700000 individuals of European ancestry. Hum Mol Genet.

[57] You D, Wang D, Wu Y, Chen X, Shao F, Wei Y, Zhang R, Lange T, Ma H, Xu H (2022). Associations of genetic risk, BMI trajectories, and the risk of non-small cell lung cancer: a population-based cohort study. BMC Med.

[58] Zhou W, Liu G, Hung RJ, Haycock PC, Aldrich MC, Andrew AS, Arnold SM, Bickeböller H, Bojesen SE, Brennan P (2021). Causal relationships between body mass index, smoking and lung cancer: Univariable and multivariable Mendelian randomization. Int J Cancer.

[59] Hemani G, Zheng J, Elsworth B, Wade KH, Haberland V, Baird D, Laurin C, Burgess S, Bowden J, Langdon R (2018). The MR-Base platform supports systematic causal inference across the human phenome. Elife.

[60] Yavorska OO, Burgess S (2017). MendelianRandomization: an R package for performing Mendelian randomization analyses using summarized data. Int J Epidemiol.

[61] Chen T, Song L, Zhong X, Zhu Q, Huo J, Chen J, Tan S, Lian X (2023). Dietary polyunsaturated fatty acids intake, air pollution, and the risk of lung cancer: A prospective study in UK biobank. Sci Total Environ.

[62] Luu HN, Cai H, Murff HJ, Xiang YB, Cai Q, Li H, Gao J, Yang G, Lan Q, Gao YT (2018). A prospective study of dietary polyunsaturated fatty acids intake and lung cancer risk. Int J Cancer.

[63] Song J, Su H, Wang BL, Zhou YY, Guo LL (2014). Fish consumption and lung cancer risk: systematic review and meta-analysis. Nutr Cancer.

[64] Zhao H, Wu S, Luo Z, Liu H, Sun J, Jin X (2022). The association between circulating docosahexaenoic acid and lung cancer: A Mendelian randomization study. Clin Nutr.

[65] Serini S, Fasano E, Piccioni E, Cittadini AR, Calviello G (2011). Dietary n-3 polyunsaturated fatty acids and the paradox of their health benefits and potential harmful effects. Chem Res Toxicol.

[66] Drouin G, Catheline D, Guillocheau E, Gueret P, Baudry C, Le Ruyet P, Rioux V, Legrand P (2019). Comparative effects of dietary n-3 docosapentaenoic acid (DPA), DHA and EPA on plasma lipid parameters, oxidative status and fatty acid tissue composition. J Nutr Biochem.

[67] Dasilva G, Pazos M, García-Egido E, Gallardo JM, Rodríguez I, Cela R, Medina I (2015). Healthy effect of different proportions of marine ω-3 PUFAs EPA and DHA supplementation in Wistar rats: Lipidomic biomarkers of oxidative stress and inflammation. J Nutr Biochem.

[68] Ikeda K, Shiraishi K, Yoshida A, Shinchi Y, Sanada M, Motooka Y, Fujino K, Mori T, Suzuki M (2016). Synchronous Multiple Lung Adenocarcinomas: Estrogen Concentration in Peripheral Lung. PLoS One.

[69] Chen KY, Hsiao CF, Chang GC, Tsai YH, Su WC, Chen YM, Huang MS, Tsai FY, Jiang SS, Chang IS (2015). Estrogen Receptor Gene Polymorphisms and Lung Adenocarcinoma Risk in Never-Smoking Women. J Thorac Oncol.

[70] Harris WS, Tintle NL, Manson JE, Metherel AH, Robinson JG (2021). Effects of menopausal hormone therapy on erythrocyte n-3 and n-6 PUFA concentrations in the Women's Health Initiative randomized trial. Am J Clin Nutr.

[71] Komal F, Khan MK, Imran M, Ahmad MH, Anwar H, Ashfaq UA, Ahmad N, Masroor A, Ahmad RS, Nadeem M, Nisa MU (2020). Impact of different omega-3 fatty acid sources on lipid, hormonal, blood glucose, weight gain and histopathological damages profile in PCOS rat model. J Transl Med.

[72] Alessandri JM, Extier A, Al-Gubory KH, Langelier B, Baudry C, LePoupon C, Lavialle M, Guesnet P (2011). Ovariectomy and 17β-estradiol alter transcription of lipid metabolism genes and proportions of neo-formed n-3 and n-6 long-chain polyunsaturated fatty acids differently in brain and liver. J Nutr Biochem.

[73] Childs CE (2020). Sex hormones and n-3 fatty acid metabolism. Proc Nutr Soc.

[74] Liu X, Peng Y, Tao R, Meng L, Li X (2022). Mendelian Randomization Study of Causal Relationship between Omega-3 Fatty Acids and Risk of Lung Cancer. Biomed Res Int.

[75] Zhuang P, Wang W, Wang J, Zhang Y, Jiao J (2019). Polyunsaturated fatty acids intake, omega-6/omega-3 ratio and mortality: Findings from two independent nationwide cohorts. Clin Nutr.

[76] Burgess S, Daniel RM, Butterworth AS, Thompson SG (2015). Network Mendelian randomization: using genetic variants as instrumental variables to investigate mediation in causal pathways. Int J Epidemiol.

[77] Tantipaiboonwong P, Chaiwangyen W, Suttajit M, Kangwan N, Kaowinn S, Khanaree C, Punfa W, Pintha K (2021). Molecular Mechanism of Antioxidant and Anti-Inflammatory Effects of Omega-3 Fatty Acids in Perilla Seed Oil and Rosmarinic Acid Rich Fraction Extracted from Perilla Seed Meal on TNF-α Induced A549 Lung Adenocarcinoma Cells. Molecules.

[78] Freitas RDS, Campos MM (2019). Protective Effects of Omega-3 Fatty Acids in Cancer-Related Complications. Nutrients.

[79] Bazan NG (2005). Neuroprotectin D1 (NPD1): a DHA-derived mediator that protects brain and retina against cell injury-induced oxidative stress. Brain Pathol.

[80] Tingö L, Hutchinson AN, Bergh C, Stiefvatter L, Schweinlin A, Jensen MG, Krüger K, Bischoff SC, Brummer RJ (2022). Potential Modulation of Inflammation by Probiotic and Omega-3 Supplementation in Elderly with Chronic Low-Grade Inflammation-A Randomized, Placebo-Controlled Trial. Nutrients.

[81] Alfano CM, Imayama I, Neuhouser ML, Kiecolt-Glaser JK, Smith AW, Meeske K, McTiernan A, Bernstein L, Baumgartner KB, Ulrich CM, Ballard-Barbash R (2012). Fatigue, inflammation, and ω-3 and ω-6 fatty acid intake among breast cancer survivors. J Clin Oncol.

[82] Chen X, Chen C, Fan S, Wu S, Yang F, Fang Z, Fu H, Li Y (2018). Omega-3 polyunsaturated fatty acid attenuates the inflammatory response by modulating microglia polarization through SIRT1-mediated deacetylation of the HMGB1/NF-κB pathway following experimental traumatic brain injury. J Neuroinflammation.

[83] Lee KH, Seong HJ, Kim G, Jeong GH, Kim JY, Park H, Jung E, Kronbichler A, Eisenhut M, Stubbs B (2020). Consumption of Fish and ω-3 Fatty Acids and Cancer Risk: An Umbrella Review of Meta-Analyses of Observational Studies. Adv Nutr.

[84] Lu Y, Chen RG, Wei SZ, Hu HG, Sun F, Yu CH (2018). Effect of omega 3 fatty acids on C-reactive protein and interleukin-6 in patients with advanced nonsmall cell lung cancer. Medicine (Baltimore).

