# Supplementary material for: Omega-6/Omega-3 Ratio as a Protective Factor in Lung Cancer: A Mendelian Randomization Study on Polyunsaturated Fatty Acids and Lung Adenocarcinoma Risk
Source: J Cancer. 2025 Jun 12;16(8):2649–62. doi: 10.7150/jca.112416 (PMC12170990; doi:10.7150/jca.112416)
Supplement: Supplementary file 1 — Supplementary materials and figures. [file jcav16p2649s1.pdf]

Supplementary Material

Supplementary Materials 1. STROBE-MR Checklist.

Supplementary Figure S2. Scatter plots of causal estimates of exposure (omega-3 fatty acids) on lung adenocarcinoma. The slope of each line corresponding to the estimated MR effect in different models, including the conventional IVW, MBE, WMM, MR-Egger, MR-RAPS and MR-PRESSO methods.

Supplementary Figure S3. Scatter plots of causal estimates of exposure (DHA) on lung adenocarcinoma. The slope of each line corresponding to the estimated MR effect in different models, including the conventional IVW, MBE, WMM, MR-Egger, MR-RAPS and MR-PRESSO methods.

Supplementary Figure S4. Scatter plots of causal estimates of exposure (omega-6 fatty acids) on lung adenocarcinoma. The slope of each line corresponding to the estimated MR effect in different models, including the conventional IVW, MBE, WMM, MR-Egger, MR-RAPS and MR-PRESSO methods.

Supplementary Figure S5. Scatter plots of causal estimates of exposure (omega-6 fatty acids to omega-3 fatty acids) on lung adenocarcinoma. The slope of each line corresponding to the estimated MR effect in different models, including the conventional IVW, MBE, WMM, MR-Egger, MR-RAPS and MR-PRESSO methods.

Supplementary Figure S6. Scatter plots of causal estimates of exposure (DHA) on lung cancer. The slope of each line corresponding to the estimated MR effect in different models, including the conventional IVW, MBE, WMM, MR-Egger, MR-RAPS and MR-PRESSO methods.

Supplementary Figure S7. Scatter plots of causal estimates of exposure (omega-6 fatty acids to omega-3 fatty acids) on lung cancer. The slope of each line corresponding to the estimated MR effect in different models, including the conventional IVW, MBE, WMM, MR-Egger, MR-RAPS and MR-PRESSO methods.

Supplementary Figure S8. Leave-one-out plot to assess if a single variant is driving the association between total omega-3 fatty acids levels and lung adenocarcinoma.

Supplementary Figure S9. Leave-one-out plot to assess if a single variant is driving the association between total DHA and lung adenocarcinoma.

Supplementary Figure S10. Leave-one-out plot to assess if a single variant is driving the association between total omega-6 fatty acids levels and lung adenocarcinoma.

Supplementary Figure S11. Leave-one-out plot to assess if a single variant is driving the association between the ratio of omega-6 fatty acids to omega-3 fatty acids and lung adenocarcinoma.

Supplementary Figure S12. Leave-one-out plot to assess if a single variant is driving the association between total DHA levels and lung cancer.

Supplementary Figure S13. Leave-one-out plot to assess if a single variant is driving the association between the ratio of omega-6 fatty acids to omega-3 fatty acids and lung cancer.

Materials 1. STROBE-MR Checklist.

Item No.	Section	Checklist item	Manuscript section and paragraph
		**Title and abstract**	
1	Title and abstract	Indicate mendelian randomization (MR) as the study's design in the title and/or the abstract if that is a main purpose of the study.	Title and abstract
		**Introduction**	
2	Background	Explain the scientific background and rationale for the reported study. What is the exposure? Is a potential causal relationship between exposure and outcome plausible? Justify why MR is a helpful method to address the study question.	Introduction, paragraphs
3	Objectives	State specific objectives clearly, including prespecified causal hypotheses (if any). State that MR is a method that, under specific assumptions, intends to estimate causal effects.	Introduction, paragraph 3
		**Methods**	
4	Study design and data sources	Present key elements of the study design early in the article. Consider including a table listing sources of data for all phases of the study. For each data source contributing to the analysis, describe the following:	Materials and methods; Figure 1
	a	Setting: Describe the study design and the underlying population, if possible. Describe the setting, locations, and relevant dates, including periods of recruitment, exposure, follow-up, and data collection, when available.	Materials and methods, "Study design" section; Figure 1

	b	Participants: Report the eligibility criteria and the sources and methods of selection of participants. Report the sample size and whether any power or sample size calculations were carried out prior to the main analysis.	Materials and methods, "Genetic instruments and data source" section
	c	Describe measurement, quality control, and selection of genetic variants.	Materials and methods, "Genetic instruments and data source" section
	d	For each exposure, outcome, and other relevant variables, describe methods of assessment and diagnostic criteria for diseases.	Materials and methods, "Genetic instruments and data source" section
	e	Provide details of ethics committee approval and participant informed consent, if relevant.	Materials and methods, "Study design" section
5	Assumptions	Explicitly state the 3-core instrumental variable (IV) assumptions for the main analysis (relevance, independence, and exclusion restriction), as well assumptions for any additional or sensitivity analysis.	Materials and methods, "Study design" section
6	Statistical methods: main analysis	Describe statistical methods and statistics used.	Materials and methods, "Genetic instruments and data source", "Mendelian randomization" and "Statistical analysis" sections
	a	Describe how quantitative variables were handled in the analyses (ie, scale, units, model).	Materials and methods, "Mendelian randomization" and "Statistical analysis" sections
	b	Describe how genetic variants were handled in the analyses and, if applicable, how their weights were selected.	Materials and methods, "Genetic instruments and data source" sections
	c	Describe the MR estimator (eg, 2-stage least squares, Wald ratio) and related statistics. Detail the included covariates and, in case of 2-sample MR, whether the same covariate set was used for adjustment in the 2 samples.	Materials and methods, "Mendelian randomization" section

	d	Explain how missing data were addressed.	N/A
	e	If applicable, indicate how multiple testing was addressed.	N/A
7	Assessment of assumptions	Describe any methods or prior knowledge used to assess the assumptions or justify their validity.	Materials and methods, "Mendelian randomization" section
8	Sensitivity analyses and additional analyses	Describe any sensitivity analyses or additional analyses performed (eg, comparison of effect estimates from different approaches, independent replication, bias analytic techniques, validation of instruments, simulations).	Materials and methods, "Mendelian randomization" section
9	Software and preregistration		
	a	Name statistical software and package(s), including version and settings used.	Materials and methods, "Statistical analysis" section
	b	State whether the study protocol and details were preregistered (as well as when and where).	N/A
		**Results**	
10	Descriptive data		
	a	Report the numbers of individuals at each stage of included studies and reasons for exclusion. Consider use of a flow diagram.	N/A
	b	Report summary statistics for phenotypic exposure(s), outcome(s), and other relevant variables (eg, means, SDs, proportions).	Supplementary Tables S1 - 7

	c	If the data sources include meta-analyses of previous studies, provide the assessments of heterogeneity across these studies.	N/A
	d	For 2-sample MR:<br><br>i. Provide justification of the similarity of the genetic variant–exposure associations between the exposure and outcome samples.<br><br>ii. Provide information on the number of individuals who overlap between the exposure and outcome studies.	N/A
11	Main results		
	a	Report the associations between genetic variant and exposure and between genetic variant and outcome, preferably on an interpretable scale.	Results, Table 1, Figure 2,3 and Supplementary Tables S8
	b	Report MR estimates of the relationship between exposure and outcome and the measures of uncertainty from the MR analysis, on an interpretable scale, such as odds ratio or relative risk per SD difference.	Results, Table 1
	c	If relevant, consider translating estimates of relative risk into absolute risk for a meaningful time period.	N/A
	d	Consider plots to visualize results (eg, forest plot, scatterplot of associations between genetic variants and outcome vs between genetic variants and exposure).	Figure 2,3 and Supplementary Figure S2-13
12	Assessment<br><br>of assumptions		
	a	Report the assessment of the validity of the assumptions.	Results, paragraph 3.1-3.3; Table 2

	b	Report any additional statistics (eg, assessments of heterogeneity across genetic variants, such as I2, Q statistic, or E-value).	Table 2
13	Sensitivity analyses<br><br>and additional analyses		
	a	Report any sensitivity analyses to assess the robustness of the main results to violations of the assumptions.	Results, paragraph 3.1-3.3; Table 2
	b	Report results from other sensitivity analyses or additional analyses.	Results, paragraph 3.1-3.3; Table 2
	c	Report any assessment of the direction of the causal relationship (eg, bidirectional MR).	Results, paragraph 3.3, Figure 3
	d	When relevant, report and compare with estimates from non-MR analyses.	N/A
	e	Consider additional plots to visualize results (eg, leave-one-out analyses).	Supplementary Figure S1-8
		**Discussion**	
14	Key results	Summarize key results with reference to study objectives.	Discussion, paragraph 1
15	Limitations	Discuss limitations of the study, taking into account the validity of the IV assumptions, other sources of potential bias, and imprecision. Discuss both direction and magnitude of any potential bias and any efforts to address them.	Discussion, paragraph 6
16	Interpretation		
	a	Meaning: Give a cautious overall interpretation of results in the context of their	Discussion, paragraph 2-5

		limitations and in comparison with other studies.	
	b	Mechanism: Discuss underlying biological mechanisms that could drive a potential causal relationship between the investigated exposure and the outcome, and whether the gene-environment equivalence assumption is reasonable. Use causal language carefully, clarifying that IV estimates may provide causal effects only under certain assumptions.	Discussion, paragraph 2-5
	c	Clinical relevance: Discuss whether the results have clinical or public policy relevance, and to what extent they inform effect sizes of possible interventions.	Discussion, paragraph 4,5
17	Generalizability	Discuss the generalizability of the study results (a) to other populations, (b) across other exposure periods/timings, and (c) across other levels of exposure.	Discussion, paragraph 6
		**Other Information**	
18	Funding	Describe sources of funding and the role of funders in the present study and, if applicable, sources of funding for the databases and original study or studies on which the present study is based.	Fundings section
19	Data and data sharing	Provide the data used to perform all analyses or report where and how the data can be accessed, and reference these sources in the article. Provide the statistical code needed to reproduce the results in the article or report whether the code is publicly accessible and, if so, where.	Data availability statement section
20	Conflicts of interest	All authors should declare all potential conflicts of interest.	Conflicts of interest section

Reference:

Skrivankova, V.W., Richmond, R.C., Woolf, B.A.R., Yarmolinsky, J., Davies, N.M., Swanson, S.A., et al. (2021). Strengthening the Reporting of Observational Studies in Epidemiology Using Mendelian Randomization: The STROBE-MR Statement. JAMA 326, 1614-1621. doi: 10.1001/jama.2021.18236

2. Supplementary Figures

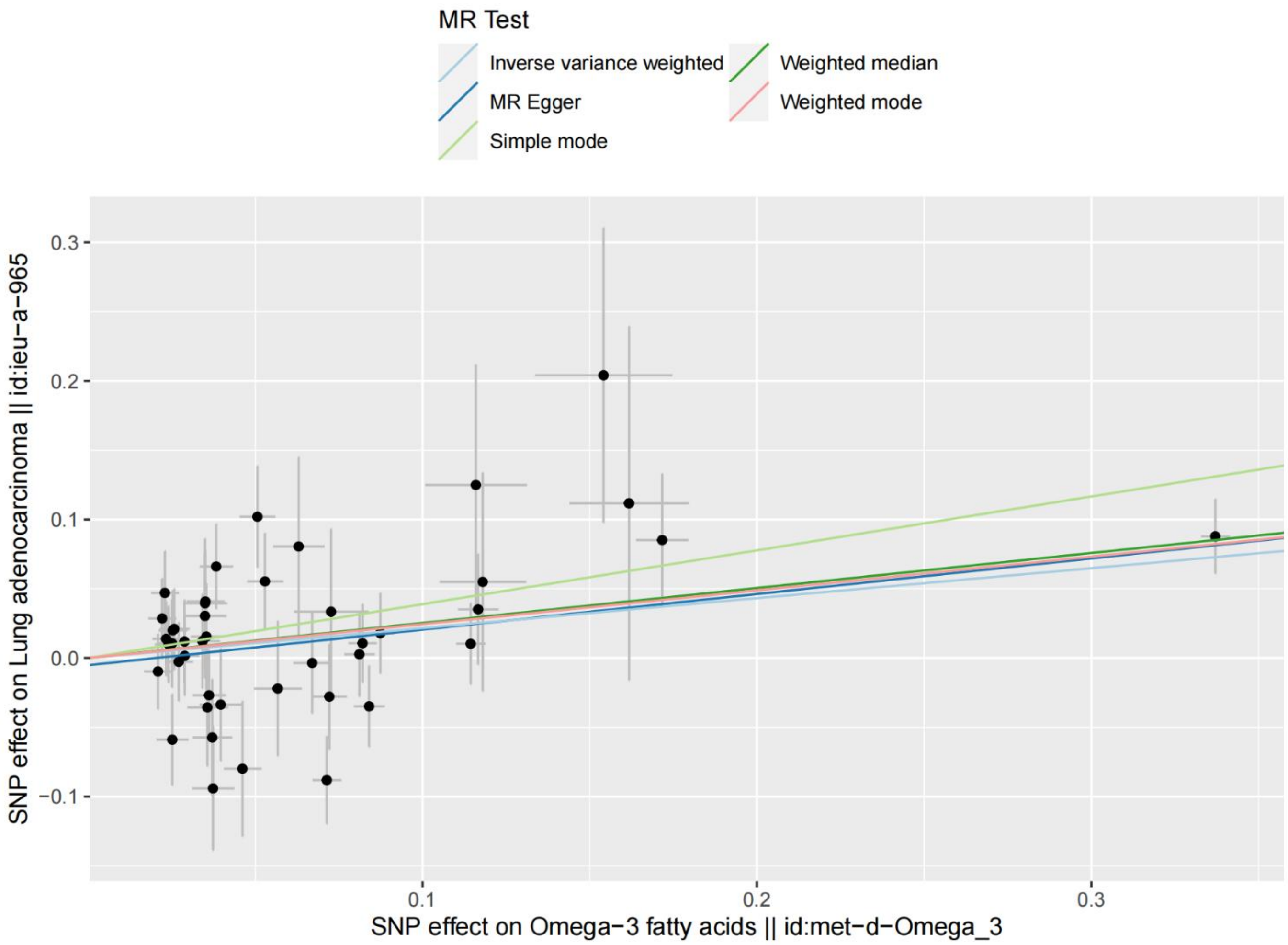

Figure S2. Scatter plots of causal estimates of exposure (omega-3 fatty acids) on lung adenocarcinoma. The slope of each line corresponding to the estimated MR effect in different models, including the conventional IVW, MBE, WMM, MR-Egger, MR-RAPS and MR-PRESSO methods.

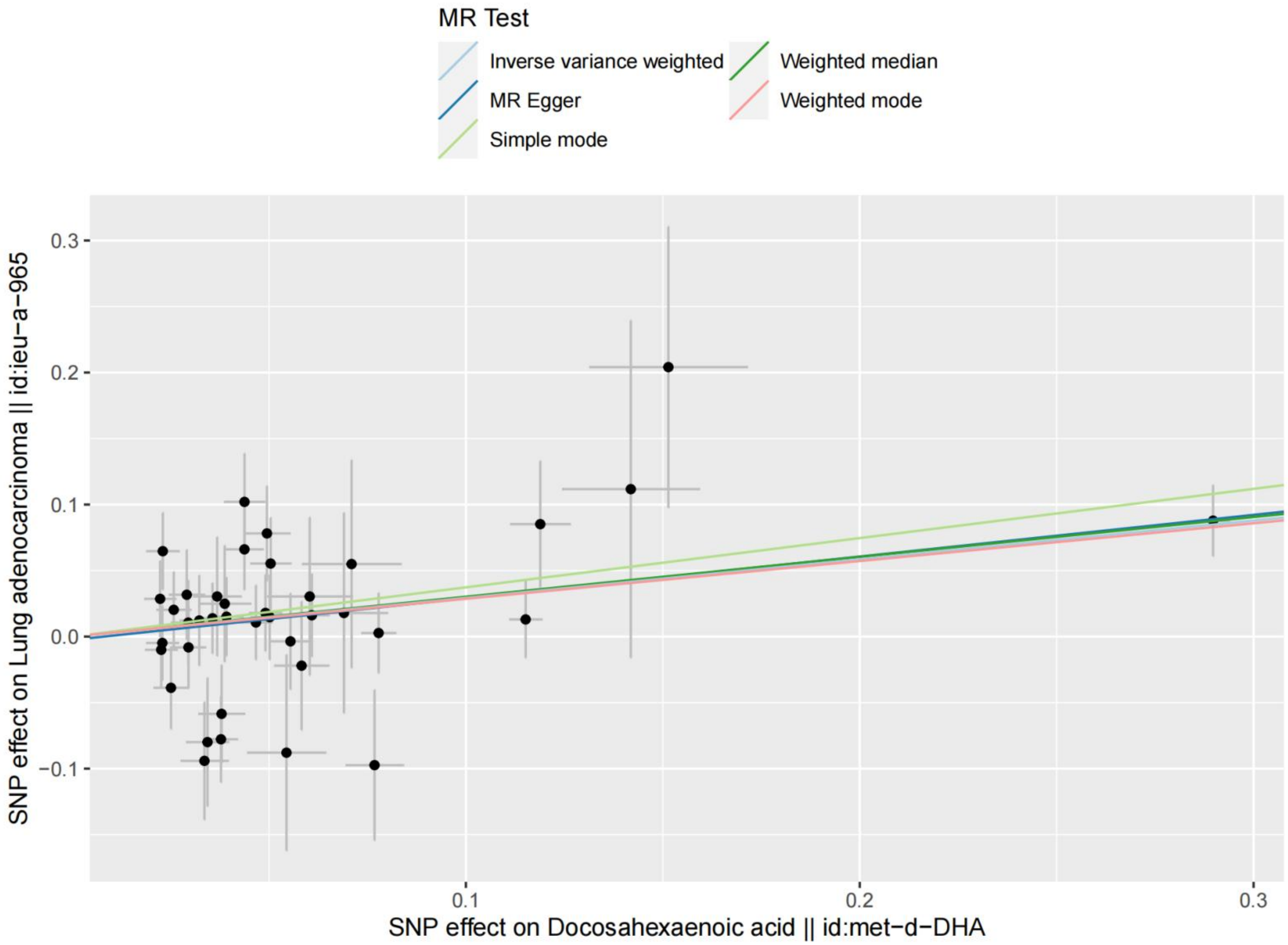

Figure S3. Scatter plots of causal estimates of exposure (DHA) on lung adenocarcinoma. The slope of each line corresponding to the estimated MR effect in different models, including the conventional IVW, MBE, WMM, MR-Egger, MR-RAPS and MR-PRESSO methods.

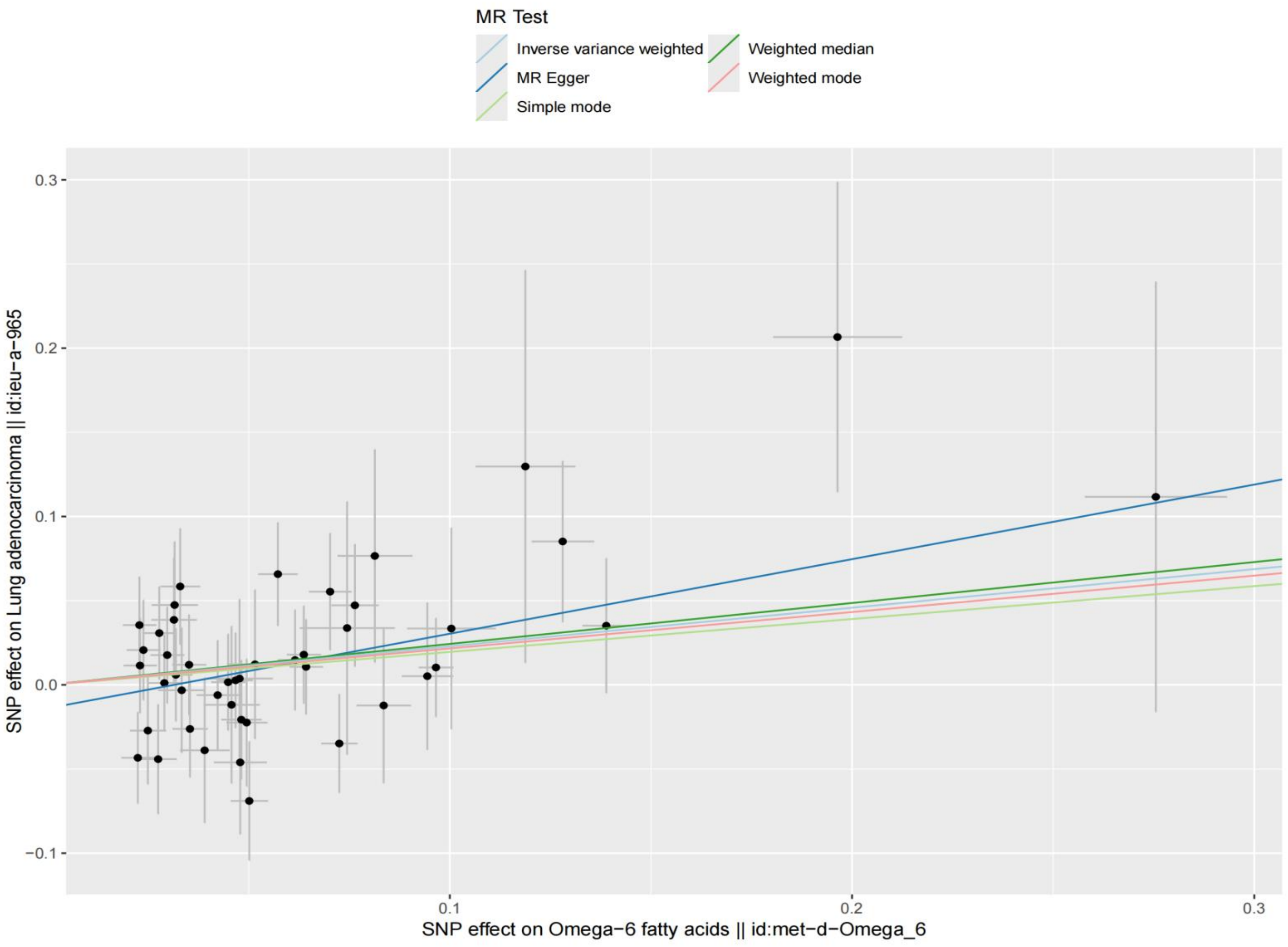

Figure S4. Scatter plots of causal estimates of exposure (omega-6 fatty acids) on lung adenocarcinoma. The slope of each line corresponding to the estimated MR effect in different models, including the conventional IVW, MBE, WMM, MR-Egger, MR-RAPS and MR-PRESSO methods.

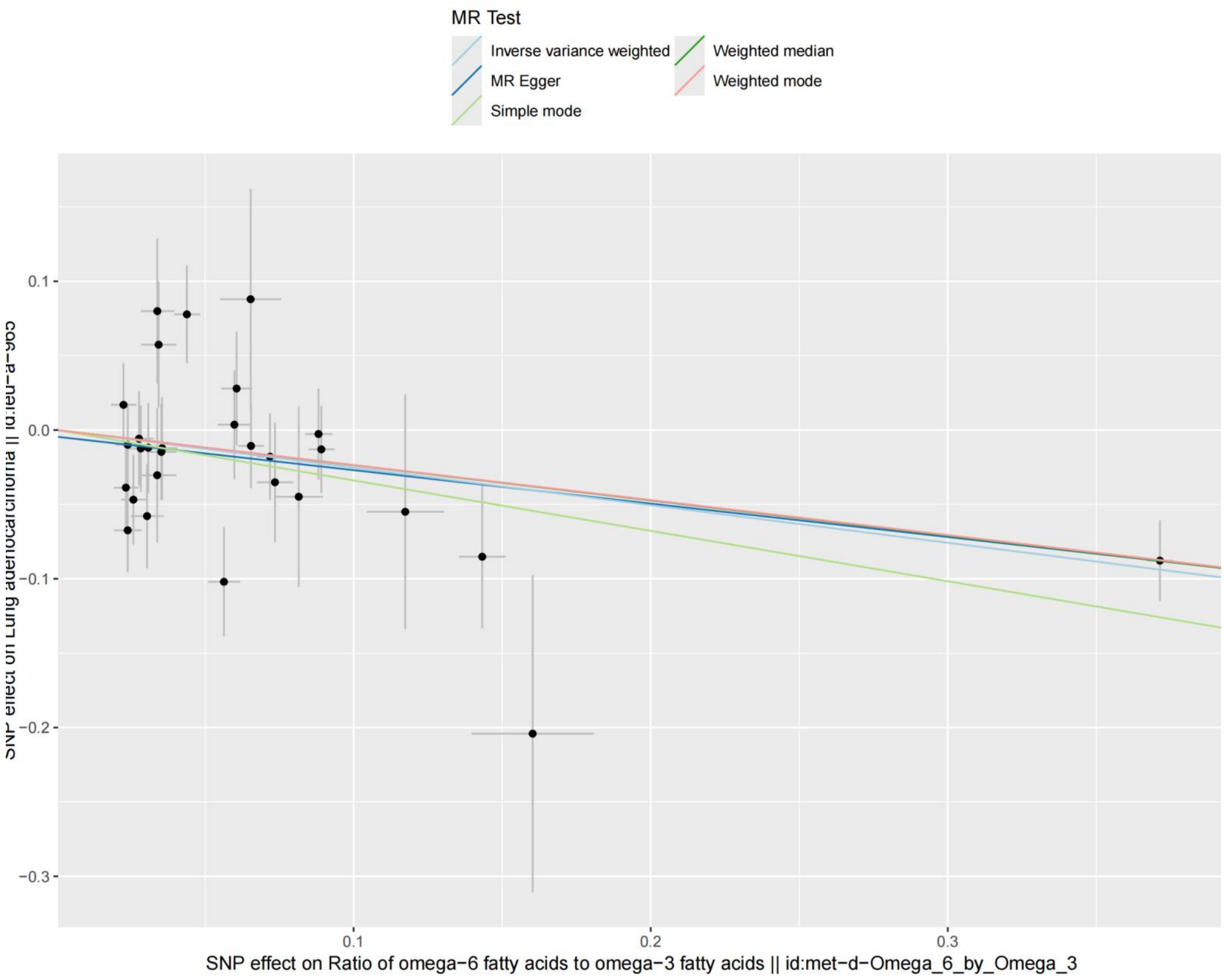

Figure S5. Scatter plots of causal estimates of exposure (omega-6 fatty acids to omega-3 fatty acids) on lung adenocarcinoma. The slope of each line corresponding to the estimated MR effect in different models, including the conventional IVW, MBE, WMM, MR-Egger, MR-RAPS and MR-PRESSO methods.

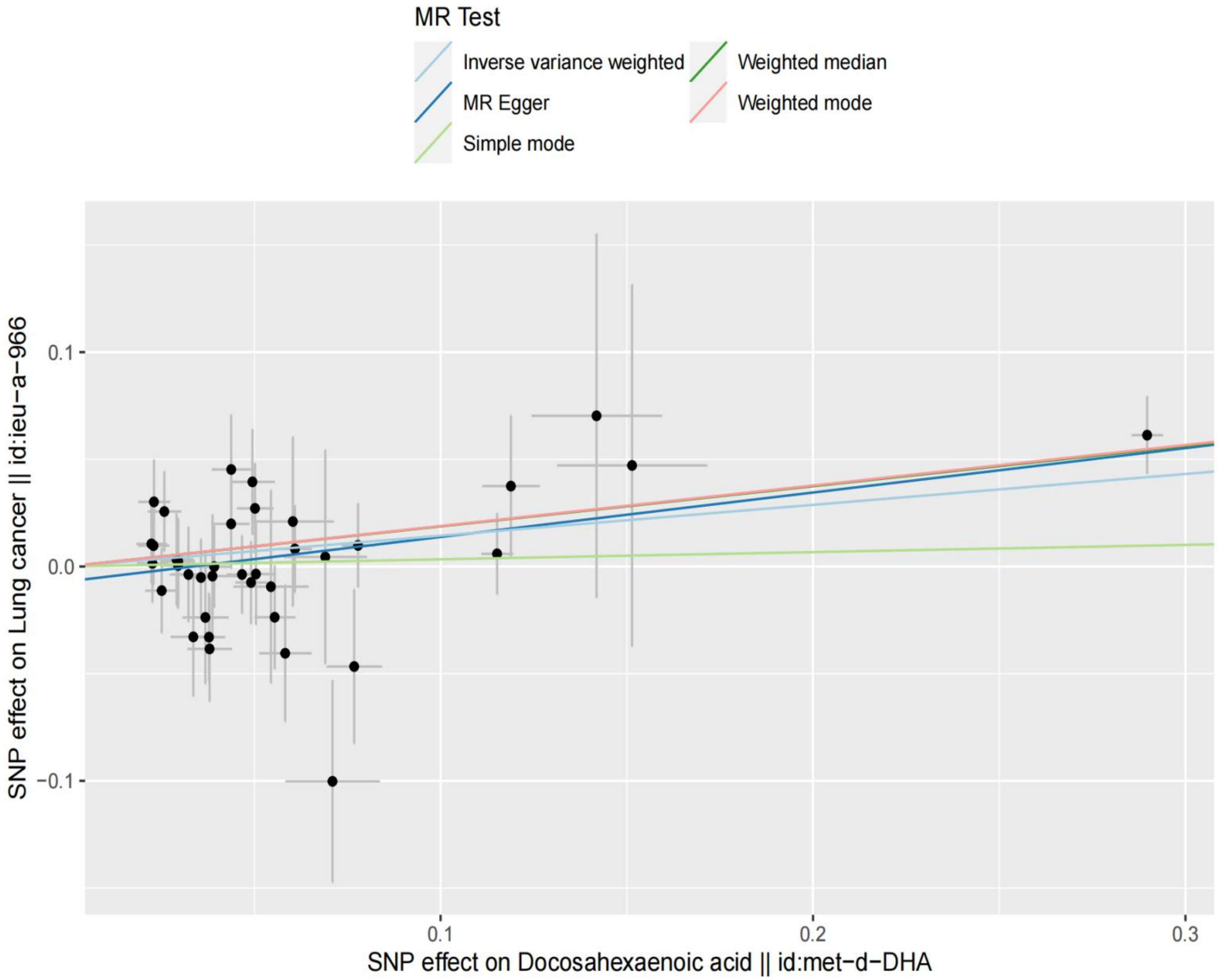

Figure S6. Scatter plots of causal estimates of exposure (DHA) on lung cancer. The slope of each line corresponding to the estimated MR effect in different models, including the conventional IVW, MBE, WMM, MR-Egger, MR-RAPS and MR-PRESSO methods.

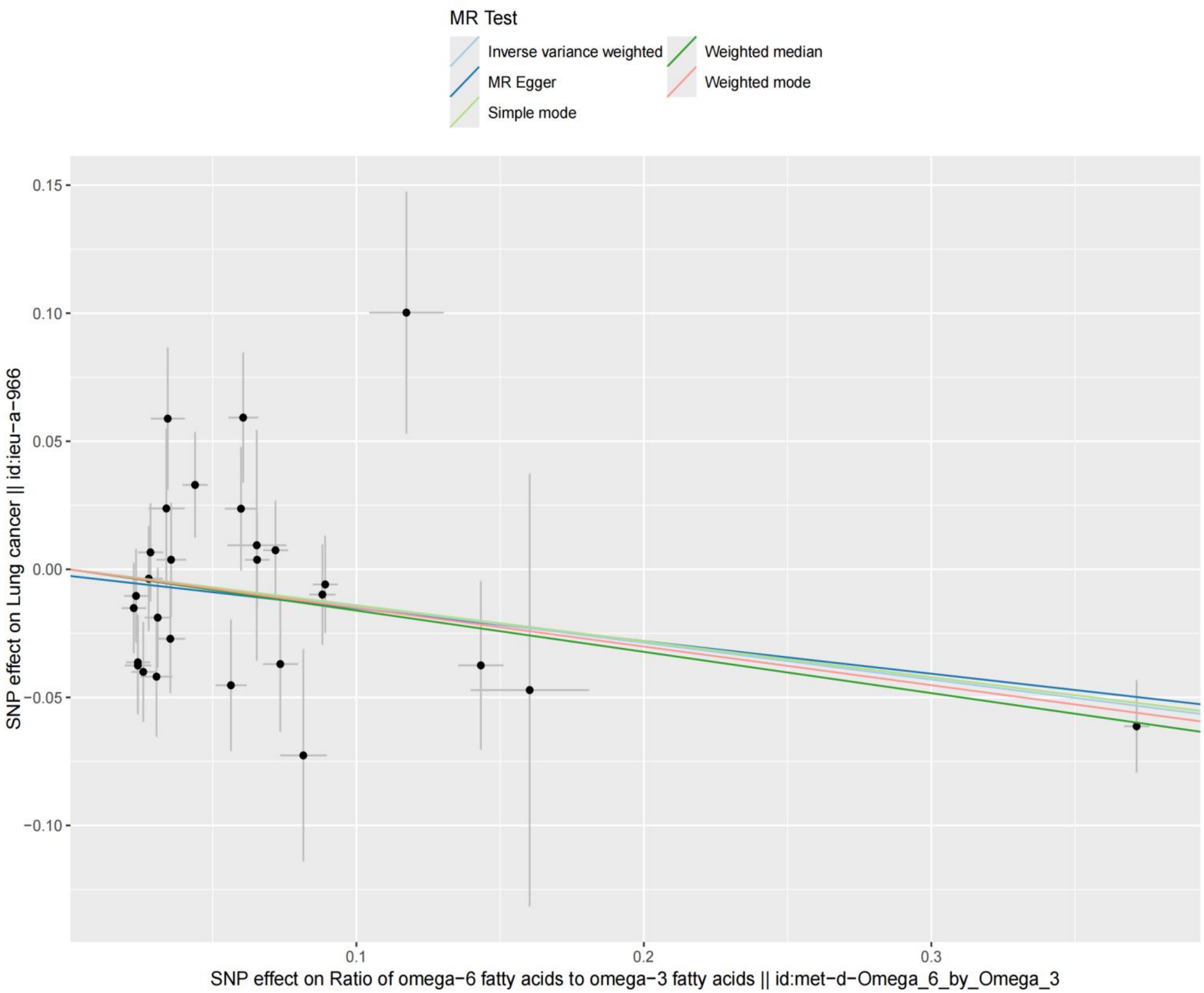

Figure S7. Scatter plots of causal estimates of exposure (omega-6 fatty acids to omega-3 fatty acids) on lung cancer. The slope of each line corresponding to the estimated MR effect in different models, including the conventional IVW, MBE, WMM, MR-Egger, MR-RAPS and MR-PRESSO methods.

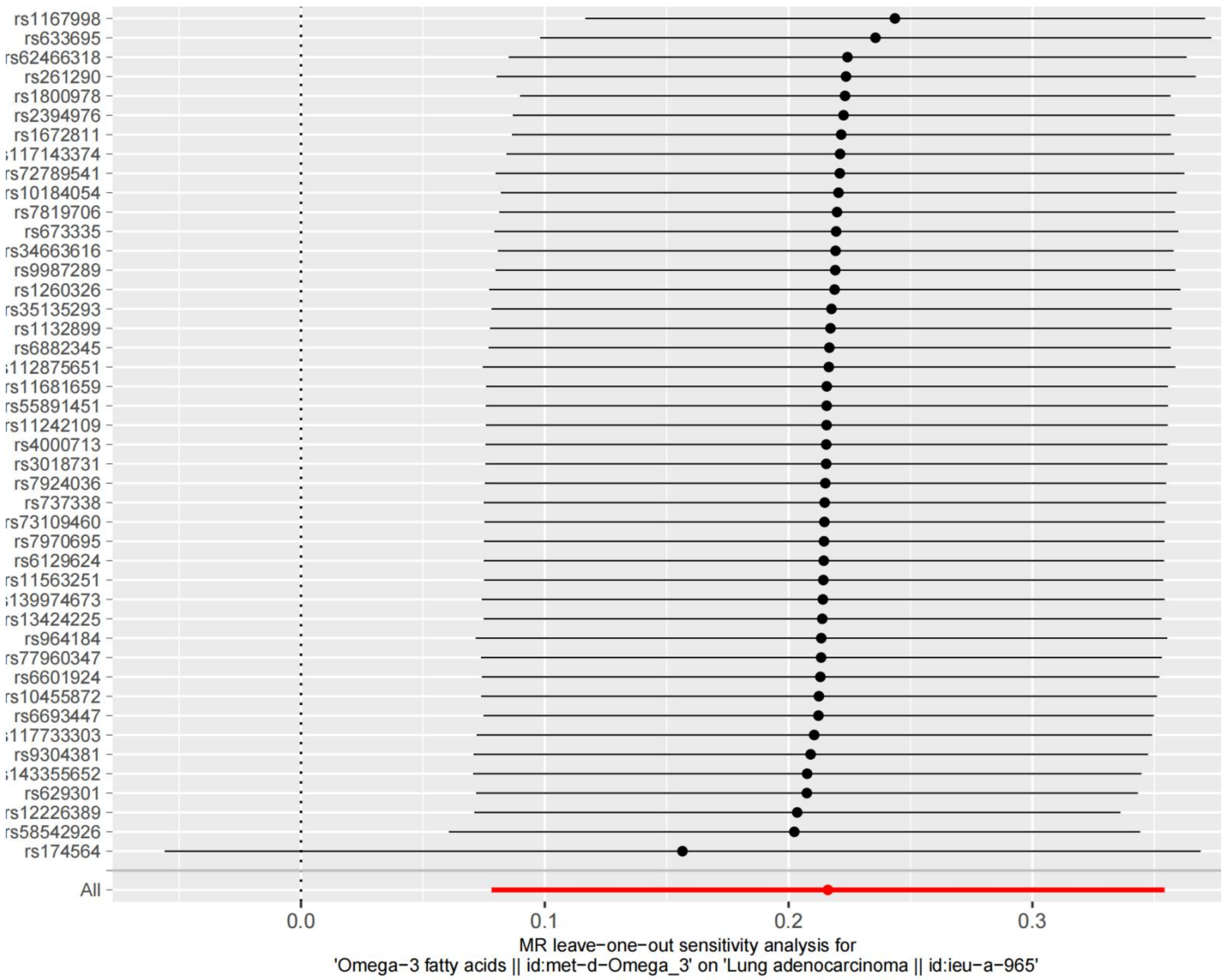

Figure S8. Leave-one-out plot to assess if a single variant is driving the association between total omega-3 fatty acids levels and lung adenocarcinoma.

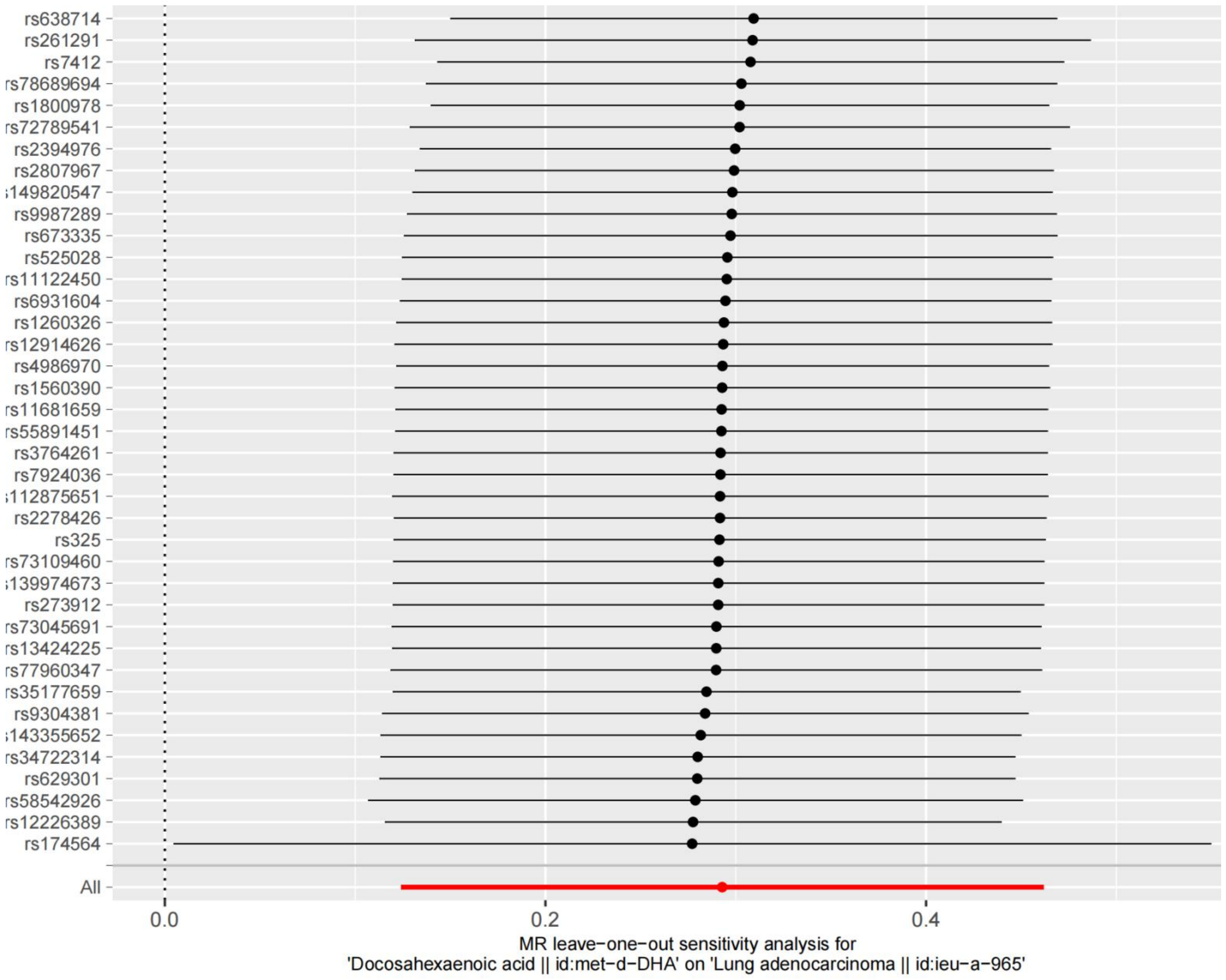

Figure S9. Leave-one-out plot to assess if a single variant is driving the association between total DHA and lung adenocarcinoma.

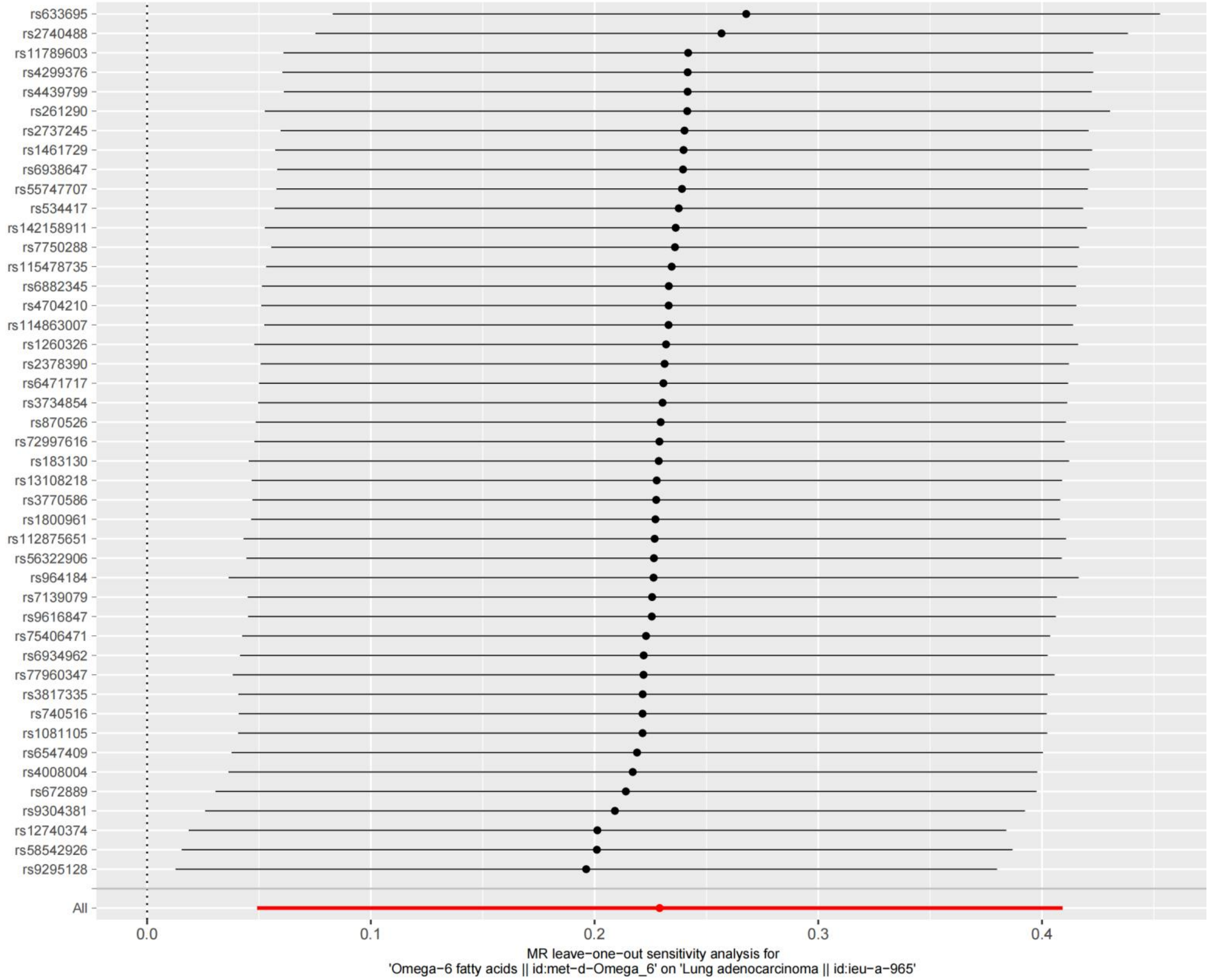

Figure S10. Leave-one-out plot to assess if a single variant is driving the association between total omega-6 fatty acids levels and lung adenocarcinoma.

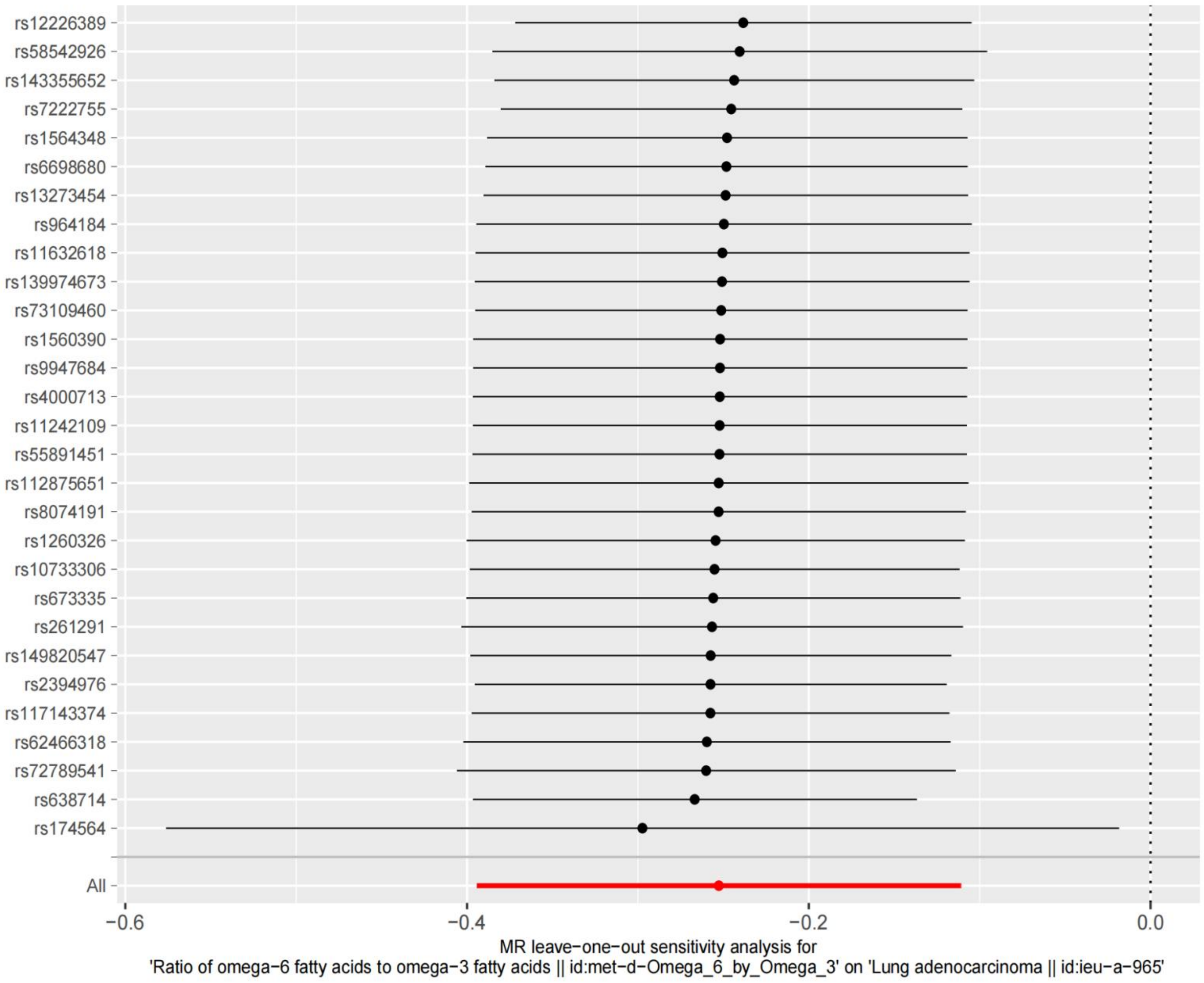

Figure S11. Leave-one-out plot to assess if a single variant is driving the association between the ratio of omega-6 fatty acids to omega-3 fatty acids and lung adenocarcinoma.

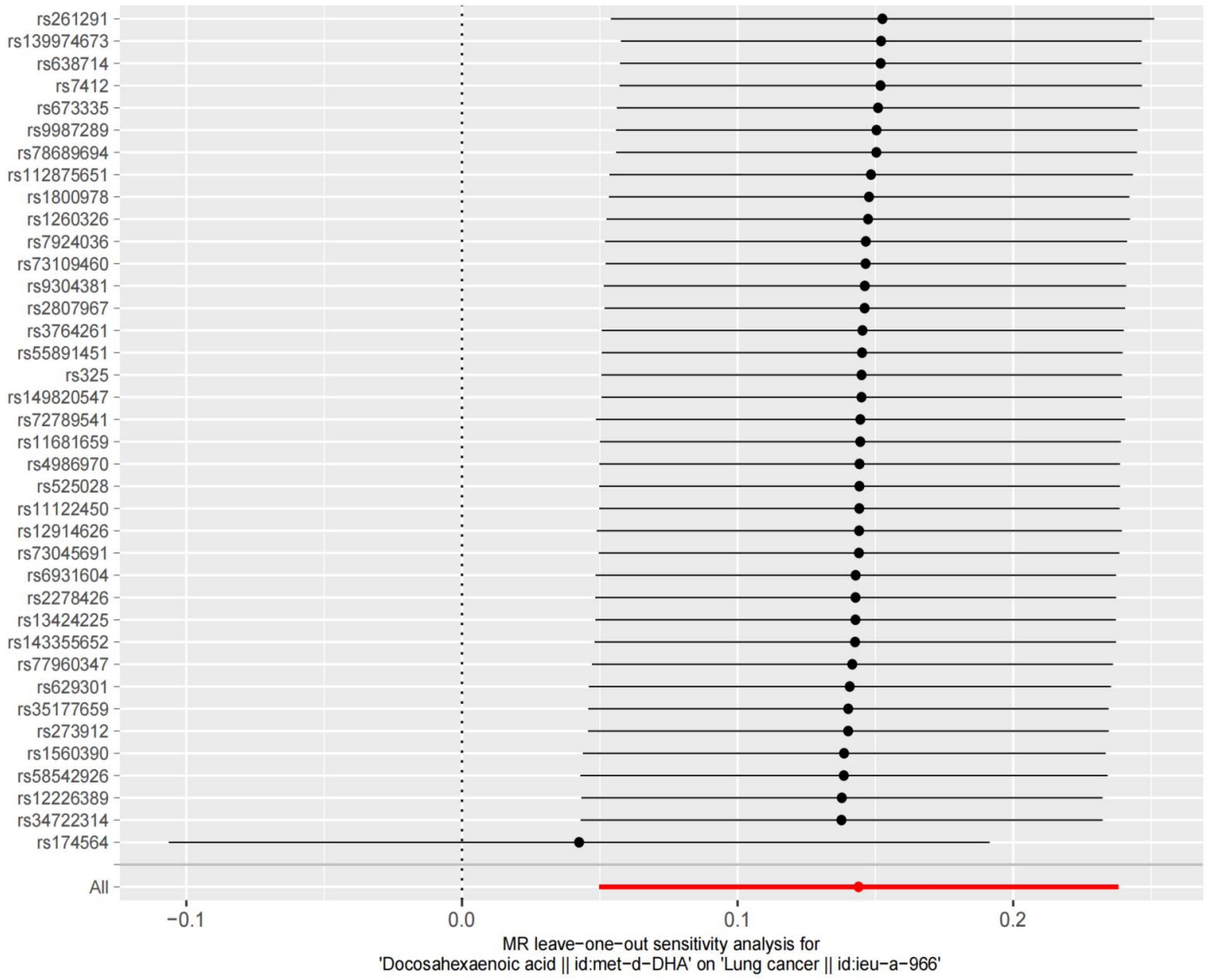

Figure S12. Leave-one-out plot to assess if a single variant is driving the association between total DHA levels and lung cancer.

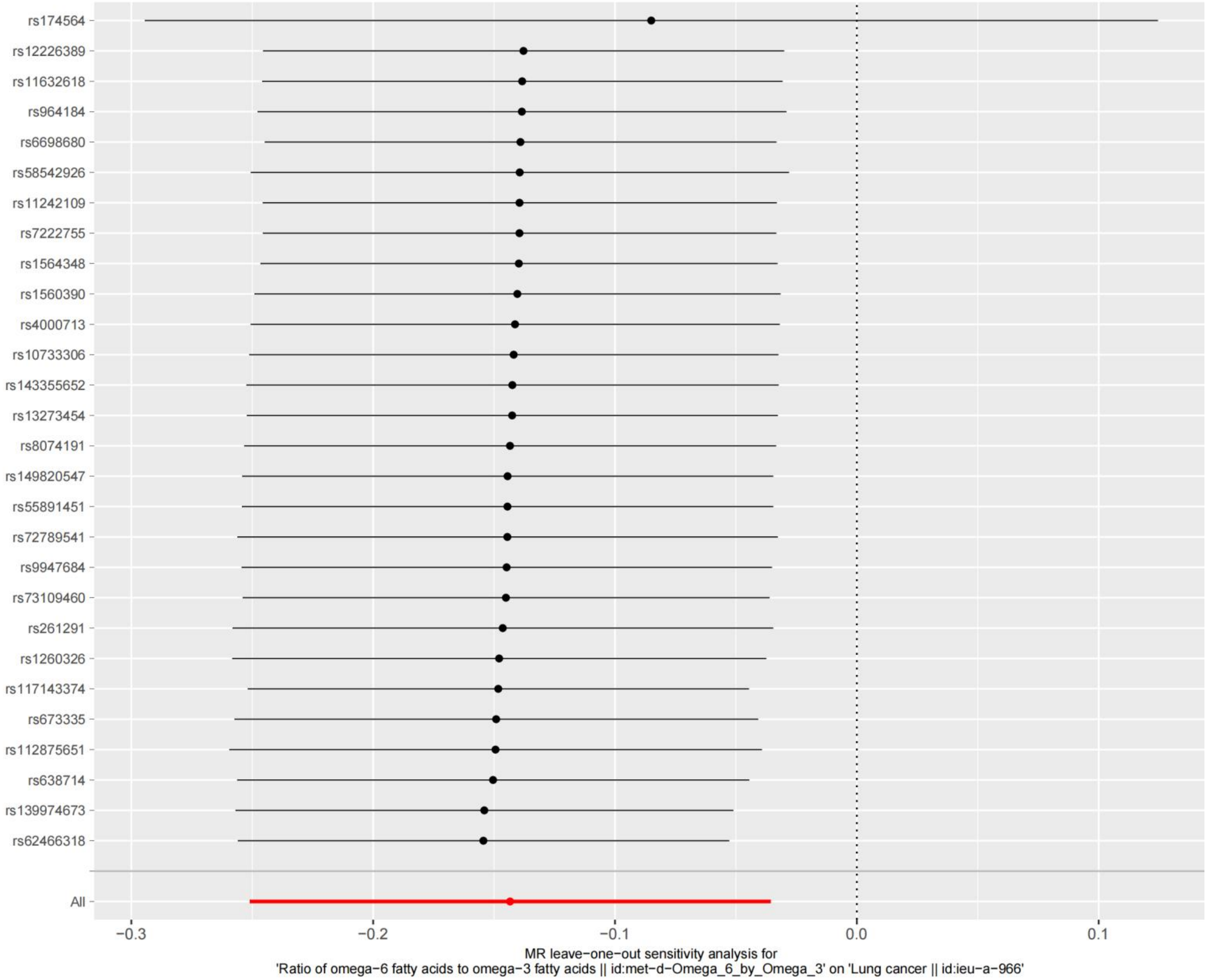

Figure S13. Leave-one-out plot to assess if a single variant is driving the association between the ratio of omega-6 fatty acids to omega-3 fatty acids and lung cancer.
